# Improved taxonomic and gene sampling advance the knowledge of deep relationships within Macrodasyida (Gastrotricha)

**DOI:** 10.1111/cla.70013

**Published:** 2025-12-16

**Authors:** Agata Cesaretti, Anush Kosakyan, Francesco Saponi, M. Antonio Todaro

**Affiliations:** ^1^ Department of Life Sciences University of Modena and Reggio Emilia Modena Italy; ^2^ National Biodiversity Future Center (NBFC) Palermo Italy; ^3^ Department of Earth and Marine Sciences University of Palermo Palermo Italy

## Abstract

Advances in Macrodasyida (Gastrotricha) phylogenetics, fuelled by new species discoveries and molecular data, are reshaping taxonomic classifications. Molecular analyses suggest polyphyly in Cephalodasyidae and Macrodasyidae, yet insufficient sampling continues to obscure precise relationships. Our study seeks to enhance the resolution of Macrodasyida's internal phylogeny through expanded taxonomic and molecular sampling. We obtained 63 new sequences from 21 Macrodasyidan species, integrating them with published data. Our dataset includes representatives from nine Macrodasyida families and 21 genera, alongside two chaetonotidans. We analysed the concatenated sequences of three genes (*18S*, *28S* rRNA, *COI* mtDNA) from 51 terminals using Maximum Parsimony, Maximum Likelihood and Bayesian Inference. Our findings confirm the polyphyly of Cephalodasyidae. *Dolichodasys* and *Paradasys* cluster with Redudasyidae, while *Cephalodasys* and *Mesodasys* form unrelated lineages. *Cephalodasys mahoae* is nested within *Paradasys* rather than *Cephalodasys*, suggesting an original misidentification. The phylogenetic placement of *Pleurodasys* remains uncertain. Macrodasyidae is non‐monophyletic, with *Urodasys* forming an independent lineage. The first molecular data ever obtained for *Dendrodasys* hint that the family Dactylopodolidae is likely polyphyletic as well. We propose an updated classification of Macrodasyida, introducing Mesodasyidae fam. nov., Urodasyidae fam. nov. and *Paraurodasys* gen. nov. Furthermore, we reassign *Dolichodasys* and *Paradasys* to Redudasyidae and *Cephalodasys mahoe* to *Paradasys*.

## Introduction

**Fig. 1:**
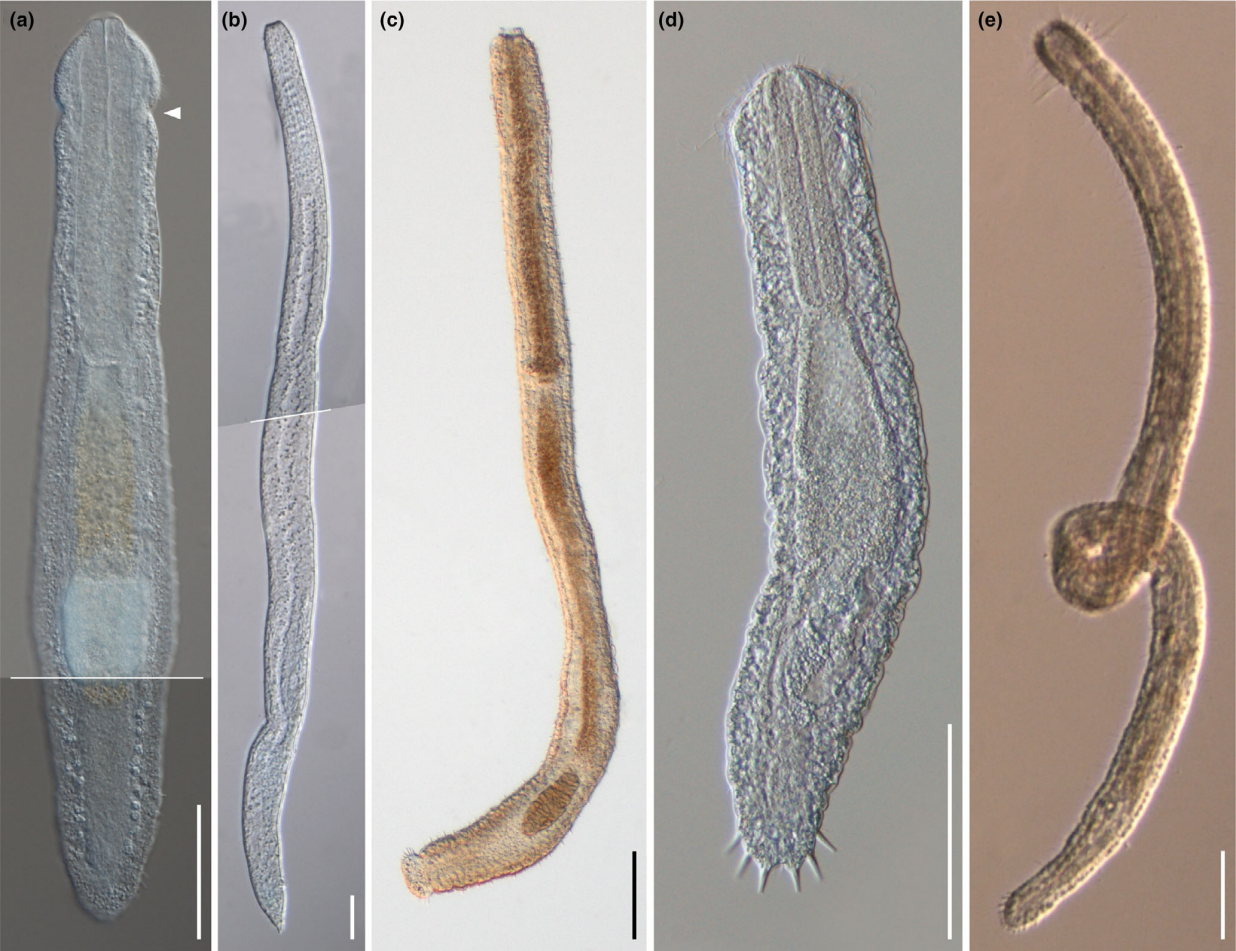
Some of the Cephalodasyidae specimens used in the study, showing the general morphology of each genus in the family. (a) *Cephalodasys maximus* Remane, 1929, photo composition, showing the anterior constriction typical of the genus (arrowhead); (b) *Dolichodasys* sp. 2, photo composition; (c) *Mesodasys laticaudatus* Remane, 1951; (d) *Paradasys* sp. 1; (e) *Pleurodasys incomptus* Todaro, Dal Zotto, Bownes and Perissinotto, 2017. Differential interference contrast microscopy (Nomarski), scale bar = 100 μm.

The phylum Gastrotricha includes microscopic, free‐living, mostly benthic invertebrates inhabiting aquatic ecosystems worldwide (Kieneke and Schmidt‐Rhaesa, 2014; Todaro et al., 2019c; Garraffoni et al., 2021). The approximately 900 formally described species are distributed into two orders: Chaetonotida (520 spp.) and Macrodasyida (380 spp.) (Saponi and Todaro, 2024; Souid et al., 2025). The taxonomy and classification of these metazoans are in a state of constant evolution, driven by the ongoing discovery of new species bearing unusual morphological traits and the increasing incorporation of molecular data into phylogenetic studies (Todaro et al., 2015, 2019b; Garraffoni and Balsamo, 2017; Kieneke and Todaro, 2021). For example, molecular phylogenetic analyses have revealed that certain taxa previously linked to traditional groups, which were primarily identified based on morphological features, occupy distinct positions on the Gastrotricha tree of life (Todaro et al., 2012, 2019b; Gammuto et al., 2024; Rataj Križanová and Vďačný, 2024; Minowa et al., 2025). This finding raises questions about the reliability of certain morphological characteristics in systematics. The morphology of Gastrotricha is not only highly diverse but also complex, making phylogenetic interpretation challenging; superficial similarities can often conceal significant underlying differences (Todaro et al., 2019b; Gammuto et al., 2024). Consequently, the phylum Gastrotricha includes some examples (see below) of what taxonomists call “systematic wastebaskets”, taxa that group species on the basis of characters that are not homologous and often on negative traits (Plotnick and Wagner, 2006; Rataj Križanová and Vďačný, 2022). The classification using plesiomorphic and negative characters results in heterogeneous and poorly defined groups, connected by superficial and often subjective similarities, without any solid evolutionary hypotheses (Plotnick and Wagner, 2006). One of the most extreme and well‐known cases of “systematic wastebaskets” is the taxonomically obsolete kingdom Protista, which was originally used to group all single‐celled eukaryotes that could not be clearly classified as animals, plants, or fungi (Whittaker, 1969). Concerning Gastrotricha, the evolutionary interpretation of the morphological characters is currently challenging due to confusion surrounding the ancestral character states at the base of different lineages. Gastrotrichs are small organisms, measuring between 80 μm and 3.8 mm in length, exhibiting a significant range of morphological diversity (Minowa et al., 2025; Souid et al., 2025), which may be even greater due to the common occurrence of unrecognized homoplasies (Rataj Križanová and Vďačný, 2022). Studying gastrotrichs can be quite difficult: they are entirely covered by a delicate cuticle, which is fragile. As a result, they are assigned to the soft‐bodied meiofauna in ecological and biogeographical studies (Artois et al., 2011; Balsamo et al., 2020; Curini‐Galletti et al., 2020). Taxonomic surveys and identification of gastrotrichs must be conducted on living specimens, as fixation processes alter their diagnostic features (Todaro et al., 2019c). This requirement makes it essential for morphological surveys to be quick and heavily reliant on the quality of the equipment used and the skills of the researchers. Additionally, re‐evaluating the original material that underpins current classifications with modern techniques is practically unfeasible. Most of the type material (holotypes) is nearly non‐existent, and descriptions prior to 1954, when the first gastrotrich photographic material was published by Wilke (1954), are complemented solely with drawings of varying quality. To address these limitations, researchers are increasingly using integrative methods to establish monophyletic groupings supported by strong phylogenetic evidence. Morphological surveys, conducted using high‐resolution microscopy techniques, such as DIC, SEM, and CLSM, are supported by detailed photomicrographs and video footages (e.g., Munter and Kieneke, 2017; Schuster et al., 2017; Campos et al., 2020; Kieneke and Todaro, 2021; Magpali et al., 2021; Cesaretti et al., 2023; Araújo, 2024). Additionally, molecular data have become crucial in enhancing traditional morphological analyses to clarify taxonomic groupings (e.g., Cesaretti et al., 2024; Saponi et al., 2024). However, many taxa remain underrepresented in molecular studies, which creates uncertainties about their origins and phylogenetic relationships. In the order Macrodasyida, the family Cephalodasyidae Hummon and Todaro, 2010 presents a puzzling example of a potential unnatural grouping. This family currently comprises five genera: *Cephalodasys* Remane, 1926 (15 species), *Dolichodasys* Gagne, 1977 (3 species), *Mesodasys* Remane, 1951 (8 species), *Paradasys* Remane, 1934 (6 species), and *Pleurodasys* Remane, 1927 (2 species) (Fig. 1).

Previously, these five genera, along with *Lepidodasys* Remane, 1926 and *Megadasys* Schmidt, 1974, were classified under the family Lepidodasyidae. However, significant morphological differences between *Lepidodasys* and the other genera, such as the presence of robust, keeled scales on the cuticle, a pharynx without pharyngeal pores, and unique characteristics in the ultrastructure of spermatozoa led Hummon and Todaro (2010) to propose a much‐needed systematic revision of the group. They determined that *Lepidodasys* should remain in the family Lepidodasyidae as its type genus, while the other genera were grouped into the newly established family Cephalodasyidae. A follow‐up study examining reproductive traits and using early phylogenetic reconstruction with molecular markers suggested transferring *Megadasys* to the family Planodasyidae (Guidi et al., 2014).

Despite the removal of *Megadasys*, the five remaining genera of cephalodasyids still only share traits that are either plesiomorphic (for example, a vermiform to strap‐shaped body and pharyngeal pores located near the junction of the pharynx and intestine) or negative traits (such as the absence of cuticular scales or spines) (Kieneke and Schmidt‐Rhaesa, 2014). Furthermore, studies using *18S* rDNA sequences suggest that this family may be polyphyletic (Todaro et al., 2012; Yamauchi and Kajihara, 2018; Kieneke and Todaro, 2021). However, the precise phylogenetic relationships of its genera remain unclear, hindering a more comprehensive revision of the taxon.

**Fig. 2:**
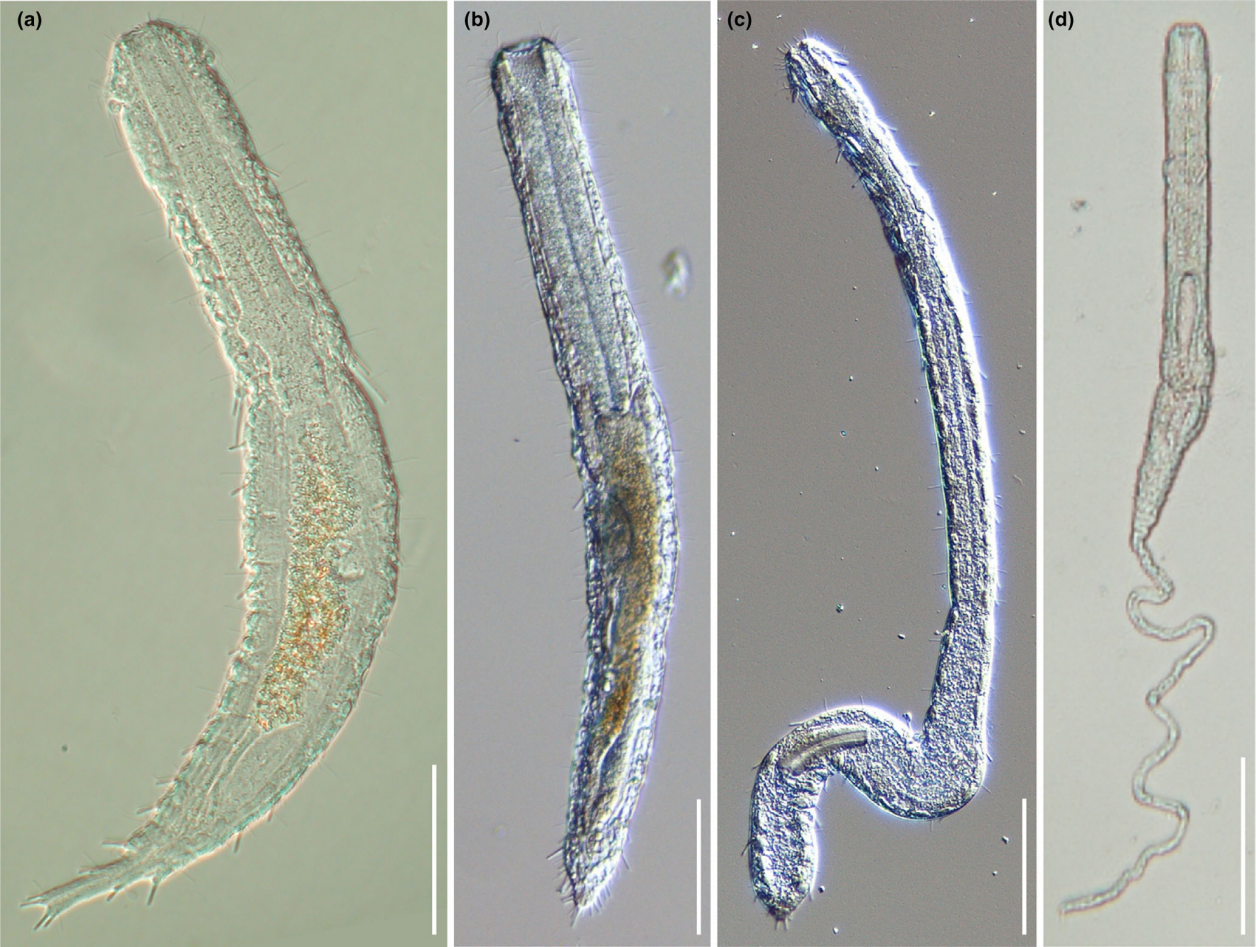
Representatives of the Macrodasyidae genera. (a) *Kryptodasys carlosrochai* Todaro, Dal Zotto, Kånneby, Hochberg, 2019; (b) *Macrodasys* sp.; (c) *Thaidasys tongiorgii* Todaro, Dal Zotto and Leasi, 2015; (d) *Urodasys completus* Todaro, Cesaretti and Dal Zotto, 2017. Differential interference contrast microscopy (Nomarski), scale bars = 100 μm. (a) Modified from Todaro et al. (2019b).

Similarly, there are indications that the family Macrodasyidae Remane, 1924 may not be monophyletic. This taxon currently comprises four genera: the well‐known *Macrodasys* Remane, 1924 and *Urodasys* Remane, 1926, along with the more recent additions of *Kryptodasys* Todaro, Dal Zotto, Kånneby and Hochberg, 2019, and *Thaidasys* Todaro, Dal Zotto and Leasi, 2015 (Fig. 2). The inclusion of the latter two genera in this family is based on a systematic approach that integrates both morphological and molecular data (Todaro et al., 2015, 2019b). In contrast, molecular phylogenetic analyses conducted thus far do not appear to support the inclusion of *Urodasys* within this family, despite significant morphological homologies that have been hypothesized to exist between *Urodasys* and *Macrodasys* (Ruppert, 1991). Furthermore, molecular phylogenetic studies have not clarified the evolutionary position of *Urodasys* within the Macrodasyida lineage (Todaro et al., 2019b; Kieneke and Todaro, 2021).

Given the challenges in interpreting morphological characteristics within a phylogenetic framework, thorough molecular investigations may help to clarify the issues mentioned above. To date, molecular research has primarily focused on a limited number of species from both Cephalodasyidae and Macrodasyidae and has often relied on sequences from just one gene. Expanding both the variety of taxa studied and the number of genes sampled is expected to resolve many of the existing uncertainties. In this study, we present new molecular data for 22 species of Macrodasyida, including 14 species from the family Cephalodasyidae. Additionally, we carried out phylogenetic analyses that incorporated sequences from five species of *Urodasys*, which were acquired only recently (Cesaretti et al., 2024). These species have not yet been part of any comprehensive phylogenetic studies focused on clarifying the internal relationships within the order Macrodasyida.

## Materials and methods

### Taxonomic and gene sampling

To explore the relationships within the Macrodasyida, which encompasses 10 families and 37 genera, we aimed to involve as many families and genera as possible in our analysis. For reliable and robust results, we based the phylogenetic analysis on the combined sequences of three molecular markers: the nuclear *18S* and *28S* rRNA genes and the mitochondrial *COI* gene. To increase/balance taxonomic representation, we focus our efforts on obtaining new molecular data from members of Cephalodasyidae (13 spp.) and other families underrepresented in previous studies or for which available molecular data are limited to a single gene such as Dactylopodololidae (1 sp.), Lepidodasyidae (3 spp.), Macrodasyidae (1 sp.), Planodasyidae, Redudasyidae (1 sp.), and Xenodasyidae (1 sp.). Unfortunately, attempts to obtain sequences from the monotypic Hummondasyidae were unsuccessful. For this study, we generated 66 new sequences from 21 species and 22 terminals. A complete list of specimens sequenced for this study is provided in Table 1. The specimens analysed in this study were collected during various faunistic surveys conducted by one of the authors (MAT). Shortly after collection, the gastrotrichs were extracted from the sandy substrate using a 7% MgCl_2_ solution; they were identified to the lowest possible taxonomic level under Nomarski optics, then fixed in 95% ethanol and stored at −20°C for further analysis. No special permissions or permits were required for collecting these organisms, as gastrotrichs are microscopic and non‐pathogenic. The field study did not involve any endangered species, and the sampling took place in publicly accessible areas. Sampled locations included the coastal areas of Costa Rica, Greece, Italy, Madagascar, South Africa, St John Island, Sweden, and Thailand (see Table 1). Additionally, we complemented our new sequences with data from 25 species sourced from GenBank (Table S1). We prioritized including species that would help establish a comprehensive taxonomic framework or whose phylogenetic relationships remain unclear, particularly those from the families Macrodasyidae and Redudasyidae. Notably, we included a significant number of *Urodasys* species (5 spp.), representing the genus's main lineages (Cesaretti et al., 2024). Sequences from these species were recently obtained in our laboratory and have never been included in comprehensive phylogenetic analyses of Macrodasyida. Intentionally, we opted to include only a limited number of species from families such as Turbanellidae and Thaumastodermatidae because their monophyletic nature is well established (e.g., Todaro et al., 2011, 2014; Kieneke and Todaro, 2021). This decision also helps to reduce the time required for our analyses. The complete dataset comprises 51 species representing nine families and 21 genera of Macrodasyida, along with two families and two genera of Chaetonotida (see Table 1, Table S1).

**Table 1:** Specimens sequenced in this study, with respective sampling areas and vouchers

Taxon	Sampling area	Voucher
**Cephalodasyidae**		
*Cephalodasys maximus* 1 Remane, 1926	Hållö Island, Sweden 58°20′27″ N; 11°12′42″ E	T2B7
*Cephalodasys maximus* 2	Hållö Island, Sweden 58°20′27″ N; 11°12′42″ E	W3B
*Cephalodasys* sp. 1	St John Island, USA 18°21′50″ N; 64°43′47″ W	W39
*Cephalodasys* sp. 2	St John Island, USA 18°21′50″ N; 64°43′47″ W	W3
*Cephalodasys* sp. 3	Ambanja district, Madagascar 13°36′52″ S; 47°53′20″ E	W10
*Cephalodasys* sp. 4	Kos Island, Greece 36°54′23″ N; 27°17′06″ E	W26
*Dolichodasys* sp. 1	Sicily, Italy 37°25′58″ N; 13°14′29″ E	T116A
*Dolichodasys* sp. 2	Umhlanga, RSA 29°43′37″ S; 31°05′24″ E	W42
*Mesodasys laticaudatus* 1 Remane, 1951	Calabria, Italy 38°54′28″ N; 16°48′43″ E	W20
*Mesodasys littoralis* 1 Remane, 1951	Sicily, Italy 36°47′18″ N; 14°29′34″ E	T111C
*Mesodasys* sp. 1	Ambanja district, Madagascar 13°36′52″ S; 47°53′20″ E	W24
*Paradasys* sp. 1	Marche, Italy 43°29′41″ N; 13°37′38″ E	M71
*Paradasys* sp. 2	Sardinia, Italy 41°03′9″ N; 8°56′16″ E	MS5
*Pleurodasys incomptus* Todaro, Dal Zotto, Bownes and Perissinotto, 2017	Umhlanga, RSA 29°43′37″ S; 31°05′24″ E	W23
**Dactylopodolidae**		
*Dendrodasys* sp. 1	Ambanja district, Madagascar 13°36′52″ S; 47°53′20″ E	W36
**Lepidodasyidae**		
*Lepidodasys martini* Remane, 1926	Hållö Island, Sweden 58°20′27″ N; 11°12′42″ E	W56
*Lepidodasys unicarenatus* Balsamo, Fregni and Tongiorgi, 1994	Tuscany, Italy 42°34′32″ N; 10°52′57″ E	W55
*Lepidodasys* sp. 1	Sicily, Italy 37°34′37″ N; 12°53′43″ E	T118C
**Macrodasyidae**		
*Thaidasys tongiorgii* Todaro, Dal Zotto and Leasi, 2015	Phuket Island, Thailand 07°48′12″ N; 98°17′55″ E	W2
**Planodasyidae**		
*Crasiella* sp. 1	Nicoya Penisula, Costa Rica 09°59′38″ N; 85°42′07″ W	W60
**Redudasyidae**		
*Anandrodasys agadasys* (Hochberg, 2003)	St John Island, USA 18°21′50″ N; 64°43′47″ W	S40
**Xenodasyidae**		
*Xenodasys riedli* (Schoepfer‐Sterrer, 1969)	St John Island, USA 18°21′50″ N; 64°43′47″ W	S42

### DNA extraction, amplification, and sequencing

Single ethanol‐fixed specimens were rinsed in clean absolute ethanol and transferred into a sterile 0.5 mL microtube using a clean, dedicated glass micropipette. The tubes with the specimens were left overnight at 25°C in a cleaned ISCO micra 18 incubator to eliminate any residual ethanol through evaporation. DNA extraction and whole‐genome amplification (WGA) were carried out using the REPLI‐g Single Cell Kit (QIAGEN®) according to the manufacturer's protocol. The presence of gastrotrich genetic material in the amplified DNA product was ascertained through a validation step involving PCR (polymerase chain reaction) amplification and Sanger sequencing of the *18S* rRNA gene of these animals. Details regarding the validation steps are available in File S1 and Table S2. All the WGA DNA products that passed the validation step were sent to the Macrogen Europe laboratories (https://www.macrogen‐europe.com/), where they were processed with a TrueSeq DNA PCR Free Library kit and *de novo* whole‐genome sequencing (WGS) on the NovaSeq 6000 Illumina Platform, generating a total of 40 million reads (paired‐ends 2 × 150 bp).

### Data assembly and gene extraction

The Illumina reads resulting from the sequencing of the WGA products were analysed through a bioinformatic pipeline, optimized from Kumar et al. (2013) (see also Serra et al., 2020; Cesaretti et al., 2024; Gammuto et al., 2024; Saponi et al., 2024). After being evaluated for quality using the Fastqc software (http://www.bioinformatics.babraham.ac.uk/projects/fastqc/) (Andrews, 2010; Kumar et al., 2018), the reads were trimmed using the Trimmomatic 0.39 software (Bolger et al., 2014), keeping parameters as default and with a minimum quality score of 30. The processed paired reads were then assembled using the SPAdes v3.6.0 software (Bankevich et al., 2012). Using the Blastn and tBlastn tools, we identified and isolated the nodes that target the ribosomal and mitochondrial regions for each sequenced specimen. The queries for the Blastn and Tblastn searches were both published gastrotrich sequences, publicly available on the NCBI GenBank database, and sequences obtained in our lab during the previous PCR validation step through Sanger sequencing. These same *18S* rRNA gene sequences were utilized as a reference to confirm the accuracy and reliability of the WGS sequencing results. This verification was performed by aligning the sequences using the BioEdit v7.2.5 Freeware software (Hall, 1999).

StructRNAfinder (https://structrnafinder.integrativebioinformatics.me/) (Arias‐Carrasco et al., 2018) and MITOS2 (Bernt et al., 2013), available on the Galaxy Europe Web Portal (https://usegalaxy.eu) (The Galaxy Community, 2024), were used to examine the nodes of interest isolated from the genomic assembly and to confirm the location of the identified genes (*18S*, *28S* and *COI*). The newly obtained COI sequences were individually analysed using the Geneious Prime software (v. 2019.2.3) (https://www.geneious.com/) to identify the correct reading frame. All the new sequences published in the present work were obtained through the WGA‐WGS pipeline.

### Phylogenetic analysis

Each gene dataset was aligned separately through the MUSCLE (multiple sequences comparison by Log expectation) algorithm (Edgar, 2004) implemented in the MEGA X software package (Kumar et al., 2018). The mitochondrial protein‐coding COI sequences were aligned as codons using the invertebrate mitochondrial genetic code. The alignments were then trimmed to the length of the majority of the sequences. The trimmed and aligned datasets resulted in 2019 (*18S* rDNA), 6988 (*28S* rDNA) and 1596 (*COI*) nucleotide sites, and the final concatenated matrix resulted in 10 603 sites. Phylogenetic analyses were conducted using the maximum likelihood (ML), Bayesian inference (BI) and Maximum Parsimony (MP) approaches. Two chaetonotidan species, *Diuronotus aspetos* Todaro, Balsamo and Kristensen, 2005 (Muselliferidae) and *Xenotrichula intermedia* Remane, 1934 (Xenotrichulidae) were chosen as the outgroup.

The ML analysis was conducted using the IQ‐TREE v.1.6.10 software (Nguyen et al., 2015; Yudina et al., 2021). The best‐fit models according to BIC (Bayesian information criterion) were determined separately for each partition by this same software: TIM3e+I+G4 for *18S* sequences, TIM3+F+I+G4 for *28S* sequences, and TVM+F+G4 for *COI* sequences. The analysis used the edge‐unlinked partition option, and was conducted using 1000 replicates of complete non‐parametric bootstrap (Guindon et al., 2010). The BI analyses were conducted in the programme MrBayes v.3.2.7 (Ronquist et al., 2012). As the evolutionary models suggested by IQ‐TREE were not available in MrBayes, we replaced them with the GTR+Γ+G4 (general time reversible) model, which is widely recognized as the best alternative in such cases (see for example, Yudina et al., 2021). This model was applied to all three partitions. The BI tree search for the final concatenated dataset ran with two parallel runs, using eight independent Markov chains over 6 000 000 generations. Tree sampling occurred every 100 generations, with a burn‐in fraction set at 25%. Convergence of the Markov chain Monte Carlo (MCMC) analyses was validated using the programmes built‐in diagnostics: the average standard deviation of split frequencies approached zero, the potential scale reduction factor (PSRF) converged to 1.00 for all parameters, and the effective sample sizes (ESS) for all parameters exceeded 200 (with a minimum ESS of 8345.943 and an average ESS of 8967.733). The MP analysis was conducted on MEGA X using 1000 bootstrap replicates and with the other parameters set as default.

All the trees were computed as unrooted and then rooted in FigTree v.1.4.3 (http://tree.bio.ed.ac.uk/software/figtree/) using *X. intermedia* and *D. aspetos* as the outgroup. Finally, the trees were edited with the CorelDraw X7 software (Corel Corporation, Ottawa, ON, Canada) to improve readability.

## Results

### Sequencing

We obtained a total of 66 new gene sequences belonging to 21 Macrodasyidan species (22 terminals), across seven families and 12 genera: Cephalodasyidae (13 species), Dactylopodolidae Strand, 1929 (1 sp.), Lepidodasyidae (3 spp.), Macrodasyidae (1 sp.), Planodasyidae Rao and Clausen, 1970 (1 sp.), Redudasyidae (1 sp.), and Xenodasyidae Todaro, Guidi, Leasi and Tongiorgi, 2006 (1 sp.).

The length of the obtained sequences varies from 1731 to 1948 bp for the *18S* rRNA gene, from 2611 to 4267 bp for the *28S* rRNA gene and from 633 to 1584 bp for the *COI* mtDNA gene. These differences are mostly imputable to limitations of the sequencing process, which produces reads of varying quality. Therefore, it was not always possible to assemble the reads containing the entire gene sequence.

### Phylogenetic analysis

**Fig. 3:**
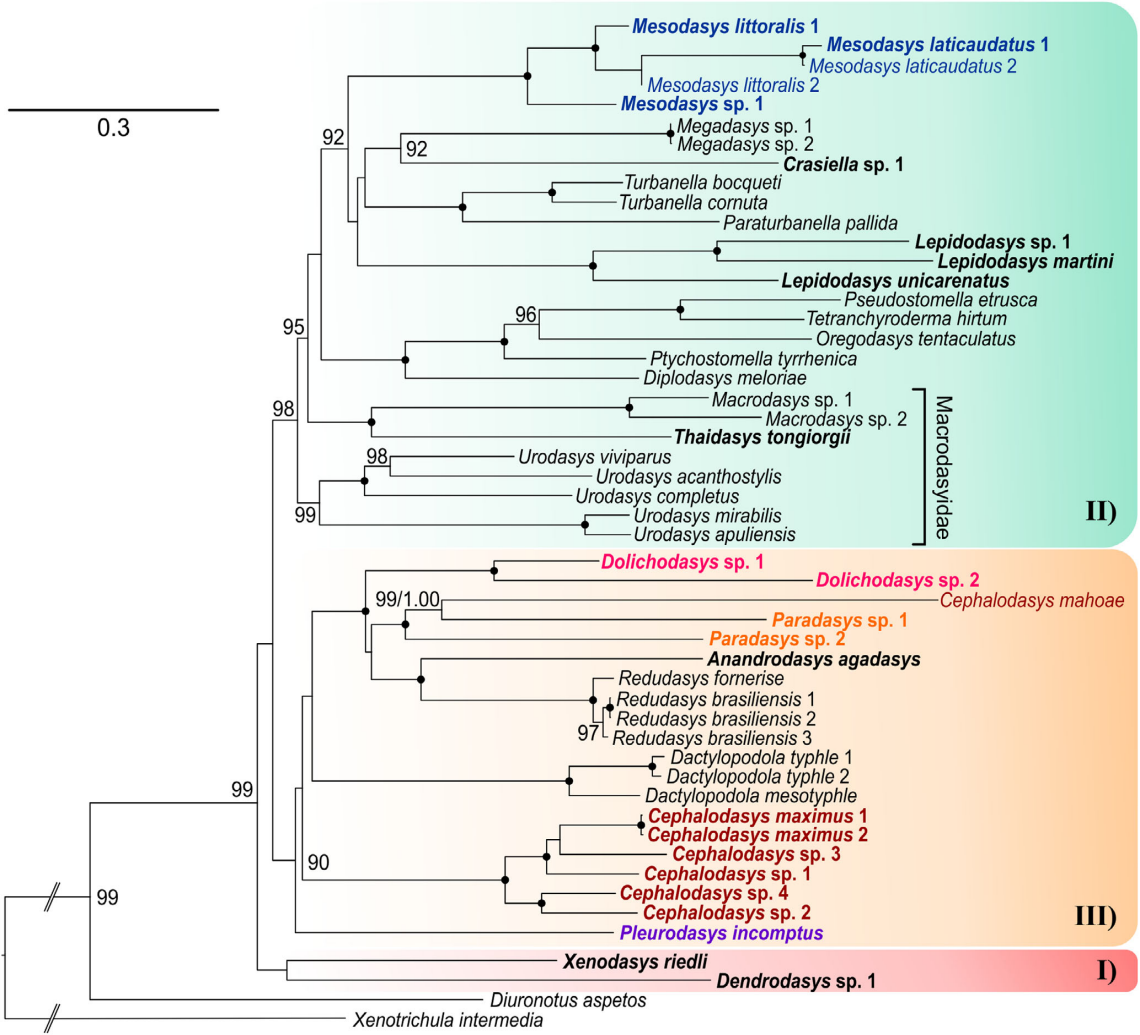
Phylogenetic relationships of the order Macrodasyida inferred from maximum likelihood (ML) analysis of concatenated *18S*, *28S* rDNA, and *COI* mtDNA sequences. The analyses include 51 terminals, of which 49 belong to the order Macrodasyida. Two Chaetonotida, *Xenotrichula intermedia* (Xenotrichulidae) and *Diuronotus aspectos* (Muselliferidae), are used as the outgroup. In bold, taxa sequenced in this study; in colour, the Cephalodasyidae coded by genus. Numbers at nodes represent bootstrap support (1000 replicates). A black dot at the node indicates full bootstrap support for the branch. Bootstrap values <90 are not reported.

**Fig. 4:**
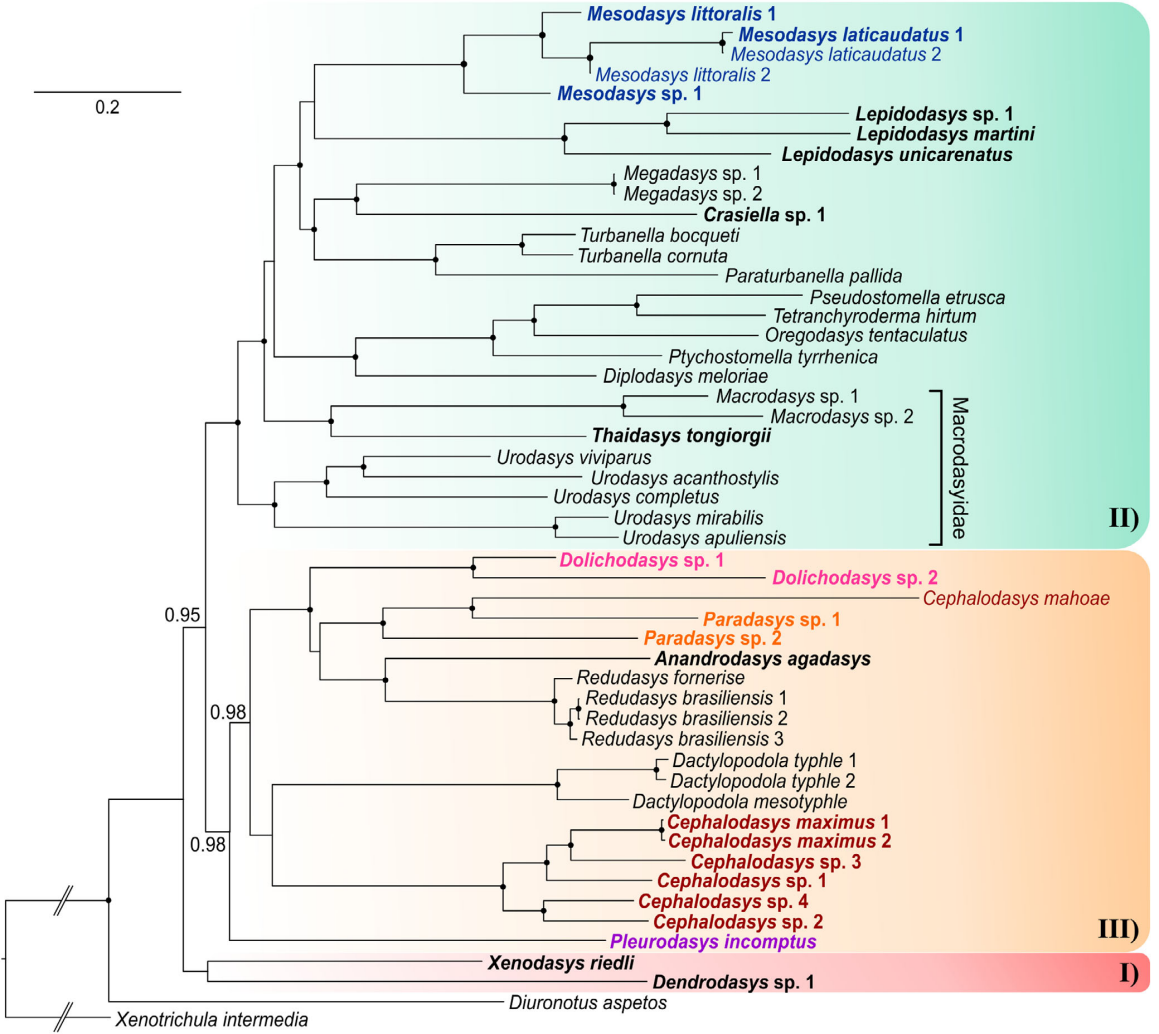
Phylogenetic relationships of the order Macrodasyida inferred from Bayesian inference (BI) analysis of concatenated *18S*, *28S* rDNA, and *COI* mtDNA sequences. The analyses include 51 terminals, of which 49 belong to the order Macrodasyida. Two Chaetonotida, *Xenotrichula intermedia* (Xenotrichulidae) and *Diuronotus aspetos* (Muselliferidae), are used as the outgroup. In bold, taxa sequenced in this study; in colour, the Cephalodasyidae coded by genus. Numbers at nodes represent posterior probability support. A black dot at the node indicates full posterior probability support for the branch. Posterior probability values <0.95 are not reported.

**Fig. 5:**
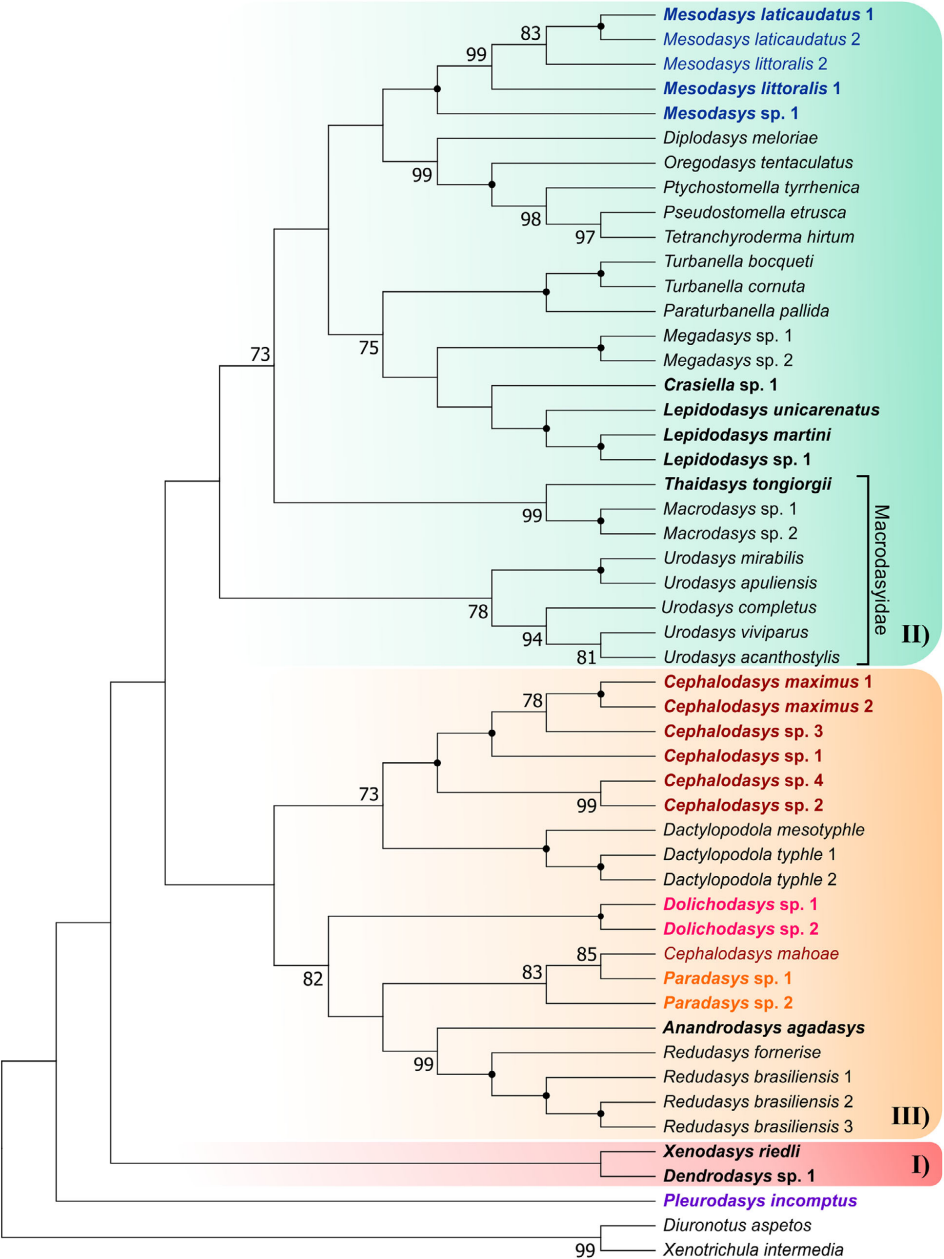
Phylogenetic relationships of the order Macrodasyida inferred from Maximum Parsimony (MP) analysis of concatenated *18S*, *28S* rDNA, and *COI* mtDNA sequences. The analyses include 51 terminals, of which 49 belong to the order Macrodasyida. *Xenotrichula intermedia* (Xenotrichulidae) and *Diuronotus aspetos* (Muselliferidae) are used as the outgroup. The most parsimonious tree resulted with length = 33 390 is shown. The consistency index was (0.287742), the retention index was (0.437808), and the composite index was 0.142973 (0.125975) for all sites and parsimony‐informative sites (in parentheses). In bold, taxa sequenced in this study; in colour, the Cephalodasyidae coded by genus. Bootstrap support for the clades is indicated in each node. A black dot at the node indicates full bootstrap support. Bootstrap values <70 are not reported. Macrodasyidan species appear distributed in three clusters (I, II, and III), except for *Pleurodasys incomptus*; see text for details.

Our phylogenetic analyses of the concatenated dataset produced trees with mostly congruent topologies. The results from both maximum likelihood (ML) and Bayesian inference (BI) analyses were consistent with each other and revealed several well‐supported main clades (Figs 3 and 4). In contrast, the Maximum Parsimony (MP) tree is less resolved at higher taxonomic levels, exhibiting lower support values at most deeper nodes. However, its general topology aligns with the other methods by resolving the analysed Macrodasyida species distributed in three main clusters (I, II, and III, Figs 3, 4, 5). In detail, genera that included two or more species were resolved as monophyletic by all analyses. The exception was *Cephalodasys*, specifically due to *C. mahoae*, which consistently clusters with *Paradasys* (Figs 3, 4, 5). Among the families represented by terminals of two or more genera, only Redudasyidae, Thaumastodermatidae, and Turbanellidae were resolved as monophyletic across all analyses. Planodasyidae was recognized as a clade by both the ML and BI analyses (Figs 3 and 4), receiving high to full support. However, the MP analysis indicated Planodasyidae as paraphyletic due to the nested position of *Lepidodasys*, with relatively low support at the nodes (Fig. 5).

The current families Cephalodasyidae, Dactylopodolidae, and Macrodasyidae were consistently found to be non‐monophyletic in all tree analyses (Figs 3, 4, 5). Specifically, Dactylopodolidae appears polyphyletic, with the early branching of *Dendrodasys* sp. along the Macrodasyida evolutionary tree. All analyses demonstrated that *Dendrodasys* sp. is associated with *Xenodasys riedli* instead of *Dactylopodola* (Cluster I); however, this grouping did not receive high support in any of the analyses (Figs 3, 4, 5).

Our results indicate that Macrodasyidae is indeed polyphyletic, with the early offshoot of *Urodasys* serving as the sister taxon to a large clade that includes other Macrodasyidae (i.e., *Macrodasys* and *Thaidasys*), as well as members from four other families (Lepidodasyidae, Planodasyidae, Thaumastodermatidae, and Turbanellidae), and terminals from the genus *Mesodasys* (LPTTM group). The alliance of *Urodasys* alongside the LPTTM taxa (Cluster II) received high to full statistical support from both the ML and BI analyses but showed relatively weak support (59% bootstrap) from the MP analysis. The internal topology of the genus *Urodasys* reveals that hermaphroditic species lacking accessory sexual organs, namely, *U. apuliensis* and *U. mirabilis*, form a separate branch, which is strongly supported in all analyses (Figs 3, 4, 5). In contrast, the parthenogenetic species *U. viviparus* is positioned within the branch that includes hermaphroditic species equipped with a sclerotized stylet, specifically *U. acanthostylis* and *U. completus*.

As initially suspected, the family Cephalodasyidae appears to be polyphyletic based on our analyses. Members of its currently affiliated genera are scattered along the Macrodasyida phylogenetic tree (Figs 3, 4, 5). The well‐sampled genera *Cephalodasys* (excluding *C. mahoae*) and *Mesodasys* emerge as distinct and phylogenetically distant lineages; however, there is no consensus across the three analyses regarding which taxon may be their closest relatives. Notwithstanding, *Cephalodasys* appears to be phylogenetically closer than *Mesodasys* to most members of Cephalodasyidae (Cluster III, Figs 3, 4, 5). The Maximum Parsimony (MP) analysis shows the strongest support for a grouping that includes *Cephalodasys* and *Dactylopodola* (73%; Fig. 5). A similar close relationship between *Cephalodasys* and *Dactylopodola* is observed in the tree from Bayesian inference, although this finding has low support (Fig. 4). Conversely, the maximum likelihood (ML) analysis suggests that *Cephalodasys* is the sister taxon to a larger group that comprises *Dactylopodola*, Redudasyidae, and two genera of Cephalodasyidae: *Dolichodasys* and *Paradasys* along with *Cephalodasys mahoae* (DRDP group). Both ML and Bayesian inference approaches support the hypothesis that the cephalodasyid *Pleurodasys incomptus* is the sister taxon to the DRDP taxa.

Our analyses reveal a clear distinction for *Mesodasys* among the cephalodasyids, positioning it in a prominently derived position within Cluster II (refer to Figs 3, 4, 5). Both ML and BI analyses consistently show that *Mesodasys* shares a closer phylogenetic connection with Lepidodasyidae, Turbanellidae, and Planodayidae than with Thaumastodermatidae, Macrodasyidae, and *Urodasys*. The ML findings strongly support (92% bootstrap support) *Mesodasys* as the sister taxon to these closely related families, while the BI analysis intriguingly suggests a potential clustering between *Mesodasys* and *Lepidodasys* (0.87 PP support). This compelling evidence underscores the unique evolutionary significance of *Mesodasys* within the Macrodasyidan lineage.

## Discussion

Our research has substantially expanded the available molecular data, particularly for members of the family Cephalodasyidae. Before our work, public repositories included the *18S*, *28S*, and *COI* gene sequences from vouchered specimens of only three species: *C. mahoae* (Yamauchi and Kajihara, 2018), *Mesodasys laticaudatus*, and *Mesodasys littoralis* (Remane, 1951), as detailed in Table S1. Notably, we acquired sequences for three genetic markers for species within the genera *Dolichodasys*, *Paradasys*, and *Pleurodasys*, for which only the *18S rDNA* sequence was previously accessible. This also applies to species in the genera *Thaidasys* (fam. Macrodasyidae), *Crasiella* (fam. Planodasyidae), *Anandrodasys* (fam. Redudasyidae), and *Xenodasys* (fam. Xenodasyidae). Additionally, we substantially increased the molecular information available for the family Lepidodasyidae by providing a complete set of sequences for three species of *Lepidodasys*. Finally, this study presents the first nucleotide sequences obtained for the previously elusive genus *Dendrodasys* Wilke, 1954 (fam. Dactylopodolidae). Our study sheds new light on the origin and evolutionary relationships of Macrodasyida, revealing important insights that challenge previous understandings. We conducted three cladistic analyses using maximum likelihood (ML), Bayesian inference, and Maximum Parsimony (MP) approaches, which yielded topologies that are highly congruent with one another. Most of the common groups received strong support, evidenced by bootstrap values and Bayesian posterior probabilities exceeding 75% and 0.98, respectively (Figs 3, 4, 5). Among the robustly supported monophyletic groups, we identify the remarkably uniform families Turbanellidae and Thaumastodermatidae, alongside most genera that align seamlessly with evolutionary hypotheses derived from morphological studies (e.g., Hochberg and Litvaitis, 2001; Kieneke et al., 2008a). However, one intriguing exception emerges: *Cephalodasys*. Notably, the Japanese species, *C. mahoae*, clusters with members of the genus *Paradasys*, revealing a critical discrepancy as it does not nest with its congeneric relatives. This unexpected finding suggests a likely misidentification, urging a re‐evaluation of previously held classifications.

### Reassignment of *Cephalodasys mahoae* Yamauchi and Kajihara, 2018 to the genus *Paradasys* Remane, 1924

During the formal description of *C. mahoae*, the authors Yamauchi and Kajihara (2018) expressed some uncertainty regarding its classification due to the striking similarity of the Japanese species to *Paradasys subterraneus* Remane, 1934. However, they ultimately based their classification decision on the results of their phylogenetic analysis, based on molecular data, which showed the new species allied with *Cephalodasys* sp. from the White Sea (Petrov et al., 2007). Additionally, *C. mahoae* features ventrolateral adhesive tubes, a characteristic previously undocumented in *Paradasys* (Hummon, 1974, 2008), but common in *Cephalodasys* (Araújo, 2024).

**Fig. 6:**
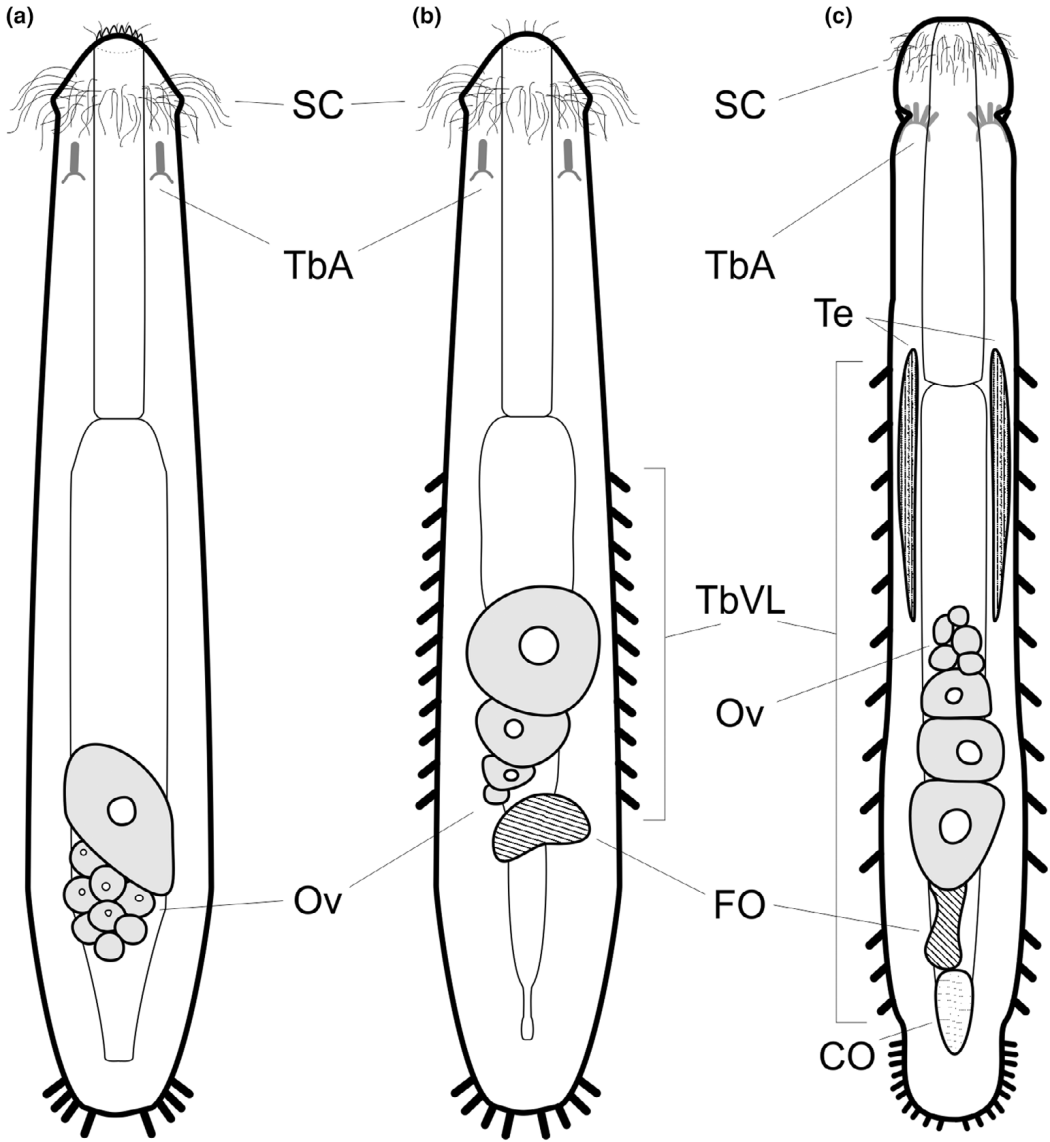
Line drawings comparing some anatomical traits of *Paradasys subterraneus* (a), *Cephalodasys mahoae* (b), and *Cephalodasys maximus* (c), combined dorsal and internal view. Anterior adhesive tubules shown in transparency. Note the direction of oocyte maturation in the ovary, which is caudocephalic in a, b, and frontocaudal in c. CO, caudal organ; FO, frontal organ; Ov, ovaries with developing oocytes; SC, sensory cilia; TbA, anterior adhesive tubes; TbVL, ventrolateral adhesive tubes; Te, testes. (a) Redrawn from Remane (1934); (b) Redrawn from Yamauchi and Kajihara (2018); (c) Redrawn from Remane (1926).

We would like to highlight that among Macrodasyida there are other genera that include species both presenting and lacking ventrolateral adhesive tubes (e.g., *Paraturbanella*), which casts doubts on the usefulness of this trait for an above‐species classification. The ventrolateral adhesive tubes of *C. mahoae* are very short, almost resembling small papillae, and papillae‐like adhesive tubes are reported in *Dolichodasys*, whose members cluster in our analyses with *Paradasys* (Figs 3, 4, 5) indicating a potential plesiomorphy. Regarding the outcome of the phylogenetic analysis conducted by Yamauchi and Kajihara (2018), we note that in their tree, another species of *Cephalodasys, C. turbanelloides*, is positioned far from the cluster that includes *Cephalodasys* sp. and *C. mahoae*. This suggests that contamination or misidentification may affect at least some of these sequences. Problems with these sequences were noted also by Paps and Riutort (2012). Despite the original decision, the classification of *C. mahoae* has remained contentious because several characteristics distinguish it from other *Cephalodasys* species. Most notably, *C. mahoae* features a broadly triangular anterior region, with lateral lobes, instead of the round shape defined by a constriction typical of the genus *Cephalodasys*. Furthermore, the oocyte development in the Japanese species follows a caudocephalic pattern, similar to *Paradasys hexadactylus* Karling, 1954, *P. littoralis* Rao and Ganapati, 1968, and *P. subterraneus* Remane, 1934. By contrast, *Cephalodasys* species exhibit frontocaudal maturation of oocytes (Fig. 6). Based on our molecular analyses and the morphological traits mentioned, we propose formally transferring *C. mahoae* to the genus *Paradasys*. Below, we present a revision of the diagnostic characters for *Paradasys*, now including the presence of lateral adhesive tubes.

### Monophyly of Thaumastodermatidae, Turbanellidae, Redudasyidae, and Planodasyidae

The families Thaumastodermatidae and Turbanellidae were consistently found to be monophyletic across all tree analyses (Figs 3, 4, 5). This finding aligns with traditional classifications based on morphological characteristics, which have also received support from early molecular phylogenetic studies (e.g., Kieneke et al., 2008a; Todaro et al., 2011; Kieneke and Todaro, 2021). Our study also provides full support for the current family Redudasyidae, established more recently on molecular data, as well as on the re‐evaluation of the morphological characteristics of its members (*Redudasys* and *Anandrodasys*; Todaro et al., 2012). These results, combined with the monophyly of all analysed genera, strengthen our findings and suggest that other phylogenetic hypotheses, when statistically supported, are highly likely to be accurate.

Planodasyidae was recognized as monophyletic by both the ML and BI analyses (Figs 3 and 4), receiving high to full support. However, the MP analysis indicated that Planodasyidae may be paraphyletic due to the position of *Lepidodasys*. Nevertheless, the relatively low support at the *Crasiella* + *Lepidodasys* node (Fig. 5) does not rule out Planodasyidae as a monophyletic group. The reproductive system and the ultrastructure of spermatozoa provide evidence for a close phylogenetic relationship between *Megadasys* and *Crasiella* (Guidi et al., 2014) reinforcing the classification of Planodasyidae as a natural group, as resolved by previous phylogenetic analyses based on molecular information (e.g., Todaro et al., 2012). We consider Planodasyidae to be monophyletic and attribute the MP result to the limited number of terminals included in our analysis, as well as to the high levels of divergence in our molecular dataset, an aspect which is known to negatively affect the results of this approach (Bergsten, 2005). Future studies that include additional species, particularly within the genus *Crasiella*, should further support this hypothesis.

### Phylogenetic status of Dactylopodolidae

Our study consistently found that the current families Cephalodasyidae, Dactylopodolidae, and Macrodasyidae are artificial, meaning they are non‐monophyletic (see Figs 3, 4, 5). Specifically, Dactylopodolidae appears to be polyphyletic, with *Dendrodasys* sp. branching early along the Macrodasyida evolutionary tree and *Dactylopodola* included in the more derived Cluster III. Currently, Dactylopodolidae includes three genera: the traditional *Dactylopodola* (type genus) and *Dendrodasys*, along with the monotypic genus *Dendropodola*, which was added later by Hummon et al. (1993). Cladistic analyses based on morphology have found this family to be monophyletic, with *Dactylopodola* frequently resolved as the earliest macrodasyidan branch (Hochberg and Litvaitis, 2001; Kieneke et al., 2008b). However, these morphological hypotheses have never been tested by molecular studies, as previous research has examined only *Dactylopodola* species. In our opinion, the available morphological information is insufficient to support Dactylopodolidae as an unquestionable monophylum. The cosmopolitan genus *Dactylopodola* includes 12 recognized species. The general morphology and ultrastructure of some of its members are relatively well understood, although traits of the reproductive apparatus and reproductive modality remain contentious (see Ruppert, 1991 vs. Kieneke et al., 2008b). *Dendrodasys* currently consists of six species that are only known at the level of gross anatomy. The general morphology of *Dendrodasys*, characterized by a distinct head, a caudal peduncle, and cross‐striated muscles suggests it is closer to *Dactylopodola* (Kieneke and Schmidt‐Rhaesa, 2014). However, differences in their reproductive systems and the distinct appearance of spermatozoa at the light microscopy level raise doubts about the close phylogenetic relationship between these two genera (see Hummon et al., 1998; Kieneke et al., 2008b; Hummon, 2011). *Dendropodola* is poorly known; its only representative, *D. transitionalis* Hummon, Todaro and Tongiorgi, 1993, has been found only once and was described based on a single subadult specimen that exhibited a general appearance intermediate between *Dactylopodola* and *Dendrodasys* (Hummon et al., 1993). All the above underscores the need for a more thorough examination of the phylogenetic status of Dactylopodolidae. In the absence of consistent, strong support for separating *Dendrodasys* from *Dactylopodola*, we prefer to provisionally maintain the current classification unchanged.

### Polyphyly of Macrodasyidae, reassignment of *Urodasys* Remane, 1926 to Urodasyidae fam. nov. and establishment of *Paraurodasys* gen. nov.

Our findings suggest that the Macrodasyidae family is polyphyletic. The early branch of *Urodasys* is positioned as the sister taxon to a significant clade that encompasses other Macrodasyidae (OM) members (specifically *Macrodasys* sp.1 and sp.2, and *Thaidasys*), along with representatives from four additional families: Lepidodasyidae, Planodasyidae, Thaumastodermatidae, and Turbanellidae, as well as species from the genus *Mesodasys* (LPTTM group). The relationship between *Urodasys* and the clade formed by OM + LPTTM taxa (in Cluster II) received robust statistical support from both the ML (98%) and BI (100%) analyses, along with nearly 60% support from the MP approach (see Figs 3, 4, 5). Given the very high statistical support for the OM clade and the strong evidence for its sister–taxon relationship with the LPTTM taxa, the hypothesis that *Urodasys* represents a phyletic lineage independent from the other members of Macrodasyidae (and any other Macrodasyida) is highly plausible.

From a morphological perspective, members of *Urodasys* are characterized by several autapomorphic traits, including an extremely long tail, a blind intestine, and spermatozoa that lack mitochondria (Balsamo et al., 2007; Kieneke and Schmidt‐Rhaesa, 2014). These distinctive traits, along with the well‐supported molecular phylogenetic placement of *Urodasys* representatives in our analyses, justify the establishment of a new family. Therefore, we reclassify *Urodasys* from Macrodasyidae to the newly established family Urodasyidae fam. nov. Below, we provide a diagnosis for this new family as well as a revised diagnosis of Macrodasyidae.

The genus *Urodasys* is notable for its remarkable diversity in reproductive structures and strategies among its members. Currently, 17 species have been described, which exhibit five distinct combinations of reproductive methods and structures. This variety includes the only known parthenogenetic and ovoviviparous gastrotrich, several species that possess a copulatory organ equipped with a sclerotized stylet, and other species that have sperm but lack a copulatory organ (Atherton and Hochberg, 2014; Todaro et al., 2019a; Cesaretti et al., 2024). A recent phylogenetic analysis, based on molecular markers, suggested that *Urodasys* species may be divided into two subclades: one consisting of hermaphroditic species that lack a copulatory organ, and the other including species that have a copulatory organ with a sclerotized stylet, as well as species that reproduce via parthenogenesis (Cesaretti et al., 2024). A similar scenario had been reached previously by work conducted on morphological data (Todaro et al., 2019a). In the current study, we analysed a subset of the data from Cesaretti et alii in a more comprehensive taxonomic framework and using a different outgroup. Our results corroborate previous findings, indicating that hermaphroditic species lacking a copulatory organ, specifically *U. apulensis* and *U. mirabilis*, form a distinct clade. In contrast, species that possess a copulatory organ with a sclerotized stylet, namely, *U. acanthostylis* and *U. completus*, as well as the parthenogenetic species *U. viviparus*, belong to a separate clade (Figs 3, 4, 5). To better reflect the differing evolutionary patterns in the reproductive systems of these organisms within a Linnaean classification framework, we suggest to separate the current *Urodasys* species into two distinct genera. We propose the establishment of a new genus, *Paraurodasys* gen. nov., to group the species with a sclerotized stylet and those that reproduce by parthenogenesis, while the type species *U. mirabilis* and others that share similar lay‐out of the reproductive system are to be maintained in *Urodasys*. Below, we provide a diagnosis for the new genus along with an amended diagnosis for *Urodasys*. *P. viviparus* is chosen as the type species for *Paraurodasys* gen. nov. in accordance with the principle of priority (article 23 of the International Code of Zoological Nomenclature).

### Polyphyly of Cephalodasyidae, with reassignment of *Mesodasys* to Mesodasyidae fam. nov. and reassignment of *Dolichodasys* Gagne, 1977 and *Paradasys* Remane, 1934 to the family Redudasyidae

As suggested by previous phylogenetic analyses, the Cephalodasyidae family appears to be polyphyletic in our study as well. The genera currently affiliated with this family are scattered throughout the Macrodasyida phylogenetic tree (see Figs 3, 4, 5). In the Results, we previously discussed *Mesodasys*, which seems to be the most phylogenetically distant taxon among the current members of Cephalodasyidae, indicated by its placement in Cluster II (Figs 3, 4, 5). In all our analyses, the clade formed by *Mesodasys* species consistently appears distinct from the other members of the LPTTM cluster, although there is no consensus on what its sister taxon might be. Regardless, our findings do not support the inclusion of *Mesodasys* in any of the currently recognized families represented in the cluster. Moreover, the general morphological characteristics of the *Mesodasys* species, along with the structure of their reproductive system, where the vas deferens are directly connected to the copulatory organ, coupled with the unique feature of hypodermic insemination (known to occur in some species, possibly applicable to all) (e.g., Ruppert, 1991; Ferraguti and Balsamo, 1995; Fregni et al., 1999), suggest that these traits are unlikely to be among the synapomorphic characteristics that differentiate members of the distinct families Lepidodasyidae, Planodasyidae, Thaumastodermatidae, and Turbanellidae (Guidi et al., 2004; Hummon and Todaro, 2010; Todaro et al., 2011; Campos et al., 2025). Given these reasons, we propose assigning the genus *Mesodasys* to a newly established monogeneric family, Mesodasyidae fam. nov. Below, we provide a detailed diagnosis for this family.

In contrast to *Mesodasys*, the other cephalodasyid genera are all resolved in cluster III (see Figs 3, 4, 5). The genera *Dolichodasys* and *Paradasys* appear to be closely related, while *Cephalodasys* and *Pleurodasys* are identified as distinct lineages. Specifically, *Dolichodasys* is positioned as a sister group to a branch formed by *Paradasys* (which encompasses *C. mahoae*) and the family Redudasyidae (comprising *Redudasys* and *Anandrodasys*). While the relationship *Dolichodasys* + *Paradasys* + Redudasyidae is consistently and highly supported across all three analyses (refer to Figs 3, 4, 5), the close phylogenetic alliance of *Paradasys* with Redudasyidae received weak statistical support.

**Fig. 7:**
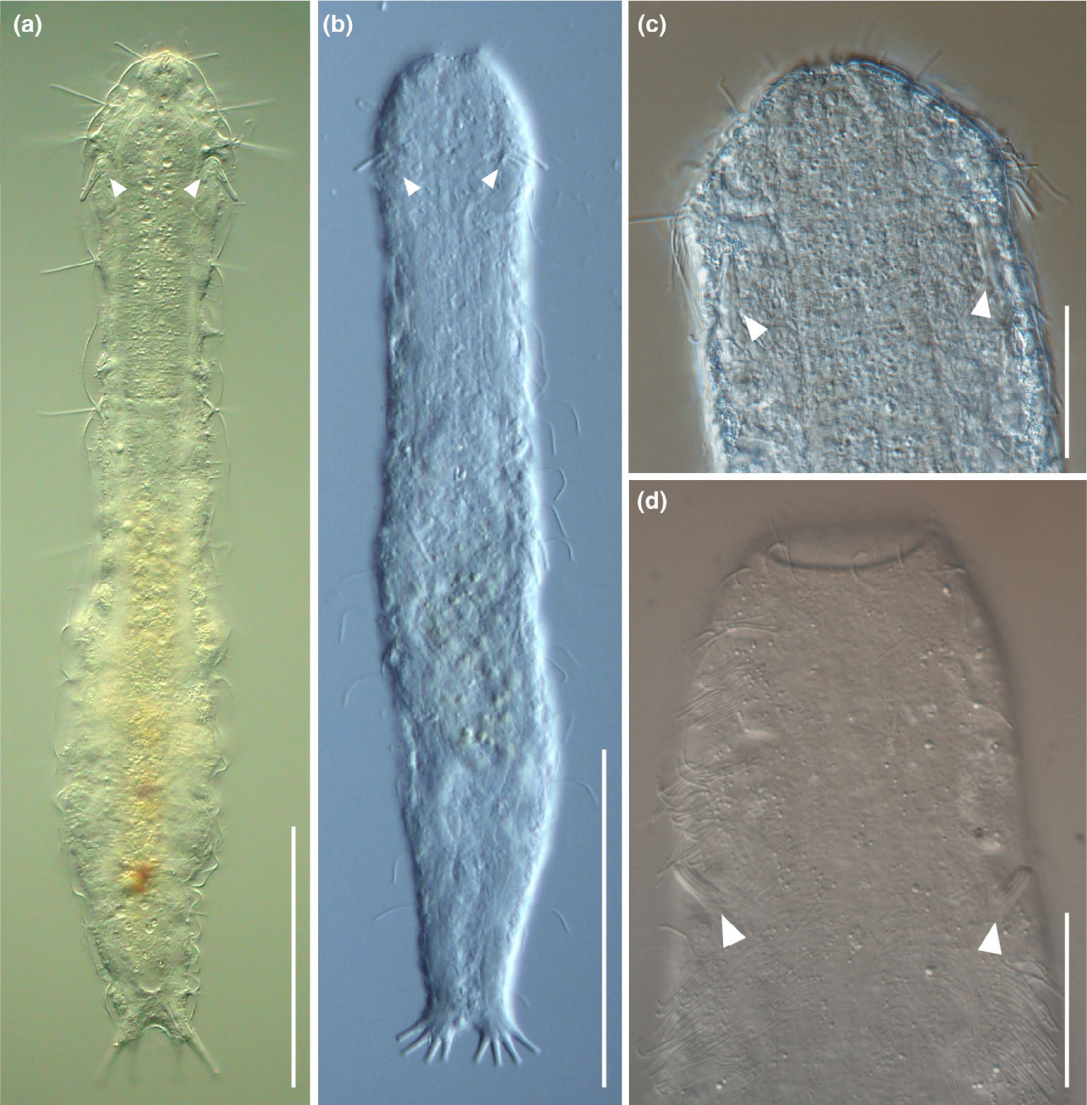
Light micrographs of representatives of the genera in the emended family Redudasyidae, ventral view showing the anterior adhesive tubes (arrowheads). (a) *Redudasys fornerise*; (b) *Anandrodasys agadasys*. (c) *Paradasys* sp. 1, anterior region; (d) *Dolichodasys* sp. 2, anterior region. Differential interference contrast microscopy (Nomarski), scale bar a, b = 100 μm; c, d = 30 μm.

We may note a trend in *Dolichodasys*, *Paradasys*, and Redudasyidae gastrotrichs towards the reduction of certain traits shared with other taxa in cluster III. For example, all gastrotrichs in cluster III bear the anterior adhesive tubes arranged in two bilateral groups. In species, such as *Dolichodasys*, *Paradasys*, and Redudasyidae, these tubes are limited to one or, at most, three per side (Fig. 7). This trend of reduction also applies to the ventrolateral tubes, which are either absent in members of *Redudasys* and most *Paradasys* or very short, resembling a papilla‐like structure in *Dolichodasys* and *C. mahoae* (*= P. mahoae*, as discussed in the present study). An exception to this trend is *Anandrodasys agadasys*, which possesses ventrolateral adhesive tubes, albeit reduced to 3–4 per side (e.g., Kieneke et al., 2013). An evolutionary trend towards reduction is also evident in the male reproductive system. The full suite of male features, including testes, sperm, and a copulatory/caudal organ, is only observed in *Dolichodasys* (Ruppert and Shaw, 1978), while the other taxa lack male features entirely. The possible exception is *C. mahoae* (= *P. mahoae*), which has been reported to contain spermatozoa in the frontal organ (Yamauchi and Kajihara, 2018). However, the nature of the sperm in this Japanese species, as seen under light microscopy, requires confirmation through ultrastructural studies, especially since their thread‐like appearance differs from the pod‐like shape of the tailless spermatozoa observed in *Dolichodasys* (Guidi et al., 2017). The results of our phylogenetic analyses, along with the morphological evidence presented above, indicate that *Dolichodasys* and *Paradasys* should no longer be classified as part of the family Cephalodasyidae. Instead, we suggest that they be included in the family Redudasyidae, which may be accomplished by slightly expanding the morphological criteria for this taxon. Consequently, we propose this reclassification and provide an updated diagnosis of the Redudasyidae in the section below.

In our analyses, the well‐sampled genus *Cephalodasys* (excluding *C. mahoae*) is recognized as a distinct lineage. Although it is phylogenetically closer to most members of the current family Cephalodasyidae than to *Mesodasys* (see Cluster III, Figs 3, 4, 5), there is no consensus among the three analyses regarding which taxon is the closest relative of *Cephalodasys*. The Maximum Parsimony analysis provides the highest support, among all analyses conducted, for a grouping that includes *Cephalodasys* and *Dactylopodola*, though the support value remains moderate (73%; see Fig. 5). A close relationship between *Cephalodasys* and *Dactylopodola* is also observed in the Bayesian inference tree, although this finding has low support (Fig. 4). From a morphological standpoint, *Cephalodasys* and *Dactylopodola* appear quite different. For instance, species of *Cephalodasys* are larger and worm‐like, while those of *Dactylopodola* are smaller and tenpin‐shaped. The posterior end of *Cephalodasys* members is unilobed, while that of *Dactylopodola* is bilobed. Other differences exist in the organization of the reproductive system and the ultrastructure of the spermatozoon (Kieneke and Schmidt‐Rhaesa, 2014). Given the lack of consensus in our phylogenetic investigation and the morphological differences, we can conclude that *Cephalodasys* and *Dactylopodola* represent two distinct evolutionary lineages, justifying their classification in two separate families within the Linnaean context.

Concerning *Pleurodasys*, the last genus currently linked to the Cephalodasyidae, our analyses reveal that the species involved in the investigation (*P. incomptus*) represents an early evolutionary branch within the order Macrodasyida. In the ML and BI trees, it is positioned as a sister taxon to the other species in cluster III. In the MP tree, it even ranks as the sister taxon of all remaining Macrodasyida (Figs 3, 4, 5). However, none of the relevant nodes received sufficient statistical support to confidently endorse one hypothesis over another. Considering the morphological similarities shared with *Cephalodasys*, such as the distinctly separated anterior region of the head from the rest of the body, the number and arrangement of the anterior adhesive tubules, and the unilobed posterior extremity, it seems reasonable to provisionally retain *Pleurodasys* within the Cephalodasyidae. Below, we propose an amended diagnosis for the Cephalodasyidae family that incorporates traits from the genera *Cephalodasys* and *Pleurodasys*.

## Conclusions

Following the reclassification of *Paradasys mahoae* comb. nov., the transfer of *Dolichodasys* and *Paradasys* to Redudasyidae and of *Mesodasys* to Mesodasyidae fam. nov., the family Cephalodasyidae now includes two genera and 16 nominal species; the family Macrodasyidae, after the transfer of *Urodasys* to Urodasyidae fam. nov., now includes three genera and 40 nominal species. Consequently, the updated order Macrodasyida includes 12 families and 38 genera.

Our results once again demonstrate that the phylogeny of Macrodasyidan gastrotrichs often deviates from the conclusions suggested so far by morphological data alone. In addition to confirming the polyphyly of the Cephalodasyidae and Macrodasyidae and finding new relationships for their former members, the multigene analysis provided new insights on the deeper phylogeny of the order Macrodasyida.

This study highlights the importance of integrating molecular data, especially through multigene sequencing when feasible, into species descriptions to enhance our understanding of their evolutionary histories. Focused efforts should be made to better describe and sequence those taxa whose position is still questionable, such as *C. miniceraus* Hummon, 1974 and *C. hadrosomus* Hummon, Todaro and Tongiorgi, 1993 in genus *Cephalodasys* (Araújo, 2024) and *P. lineatus* Rao, 1980 in genus *Paradasys*, to verify and eventually update their classification. This would both strengthen the diagnosis of each taxon, hopefully reducing the number of “systematic wastebaskets” in the phylum, and enhance the taxonomic sampling in future molecular studies on the evolution of gastrotrichs.

Including additional species from the genera *Dendrodasys* and *Pleurodasys* in the analysis could provide better insights into the phylogenetic status of the Dactylopolidae and enhance our understanding of the origin and early evolution of Macrodasyidan Gastrotricha. Several previous reconstructions of the stem species of Gastrotricha have been based on the assumption that either the genus *Dactylopodola* or *Cephalodasys* is the sister taxon to the rest of the order Macrodasyida (Kieneke and Schmidt‐Rhaesa, 2014). However, molecular data now challenge this assumption. Including a complete set of three genes from *Hummondasys jamaicensis* (Hummondasyidae) in the analysis could help clarify the origin and cause of the rare male reproductive system configuration in this species, characterized by the confluence of the vas deferens in the caudal organ. A similar configuration is also found in *Mesodasys* and in Thaumastodermatinae. However, it remains unresolved whether this similarity is due to convergent evolution, parallel evolution, or evolutionary reversals.

While the findings outlined in this article do not resolve all outstanding questions, they illuminate several critical gaps in our understanding of Macrodasyida evolution. More importantly, they lay a solid groundwork for future research endeavours. To advance our knowledge, it is essential that upcoming studies enhance molecular and taxonomic sampling in phylogenetic analyses. Additionally, there is a pressing need to delve deeper into the fascinating traits of these organisms, particularly their reproductive biology and ecological roles. Despite their diminutive size, these remarkable creatures demonstrate astonishing diversity, offering a wealth of unexplored avenues for investigation. Embracing these opportunities could significantly enrich our understanding of this unique group.

## Diagnoses

Order Macrodasyida Remane, 1925 [Rao and Clausen, 1970]

**Family Cephalodasyidae** Hummon and Todaro, 2010

**Emended diagnosis:** Elongate Macrodasyidans up to 1800 μm in total length. Body strap‐shaped, flattened ventrally and vaulted dorsally; head rounded, marked by a posterior constriction; head sensorial structures in the form of circumcephalic cilia; posterior end rounded, broadly expanded or tapering into a medial process. Cuticular covering smooth, without scales or spines; epidermal glands often present. Anterior adhesive tubes (TbA) in two groups, inserting on fleshy “hands”; dorsolateral, lateral, and ventrolateral adhesive tubes (TbDL/TbL/TbVL) arranged in columns along the body; posterior adhesive tubes (TbP) arranged marginally around the posterior end. Ventral ciliation split into two paired longitudinal bands along the body, reuniting caudally. Mouth, terminal or slightly subterminal, narrow; buccal cavity broadly cylindrical, lightly cuticularized. Sphincter muscle developed around the mouth opening; well‐developed striated radial pharyngeal musculature. Circular muscles present in lateral regions of the body. Y‐cells absent. Pharynx bearing pores at the base, opening ventrolaterally; broad anterior intestine, narrowing caudally; anus ventral. Hermaphroditic; ovary single, central, oocytes maturing posterior to anterior (*Pleurodasys*) or anterior to posterior (*Cephalodasys*). Testes paired, male gametes mature posterior to anterior. Frontal organ usually present; caudal organ occasionally present in *Cephalodasys*, absent in *Pleurodasys*. Interstitial, marine. Included genera: *Cephalodasys* Remane, 1926 (type genus); *Pleurodasys* Remane, 1927.

**Family Macrodasyidae** Remane, 1924

**Emended diagnosis:** Elongate macrodasyidans up to 1000 μm in total length. Body strap‐shaped, elongated; head bluntly rounded or ovoid, sometimes marked by a posterior constriction (*Kryptodasys*, *Thaidasys*); head sensorial structures in the form of circumcephalic cilia and pestle organs, or leaf‐like organs (*Thaidasys*); posterior end ovoidal or tapering, sometimes in the form of a short tail (*Macrodasys*). Cuticular covering smooth, without scales or spines; epidermal glands either inconspicuous or visible (*Thaidasys*). TbA 2–7 per side, inserted directly on the body surface in diagonal columns or short arcs; TbD/TbDL sometimes present; TbL/TbVL arranged in columns along the body; TbP arranged marginally around the posterior end or along the tail. Ventral ciliation either split into two paired longitudinal bands along the body (*Thaidasys*), or forming a single field that can split into two paired longitudinal bands along the caudal region. Mouth, terminal, medium size; buccal cavity lightly cuticularized, shallow. Pharynx bearing pores significantly anterior to the pharyngo‐intestinal junction, opening ventrolaterally; intestine straight, sometimes broader in the middle section (*Kryptodasys*); anus ventral. Hermaphroditic; ovary single, oocytes maturing posterior to anterior. Testes paired, elongated, starting near the pharyngo‐intestinal junction, or absent (*Thaidasys*). Frontal organ, present or absent (*Thaidasys*); caudal organ is present as a muscular organ containing a copulatory tube or a canal. Interstitial, marine. Included genera: *Macrodasys* Remane, 1924 (type genus); *Kryptodasys* Todaro, Dal Zotto, Kånneby and Hochberg, 2019; *Thaidasys* Todaro, Dal Zotto and Leasi, 2015.

**Family Mesodasyidae fam. nov**.

LSID: urn:lsid:zoobank.org:act:53A43C3C‐92C2‐446D‐8CD9‐BECC3F9172B6

**Diagnosis:** Macrodasyidans up to 2000 μm in total length. Body strap‐shaped, elongated; head bluntly rounded or truncated, unmarked from the body; head sensorial structures in the form of circumcephalic cilia; posterior end rounded, tapering, or ending in a rounded caudal lobe. Cuticular covering smooth, without scales or spines; numerous epidermal glands are visible for the whole length of the body, in lateral columns. TbA numerous, inserted directly on the body surface in transverse rows, diagonal columns, or a single uninterrupted arc following the anterior profile; TbD numerous when present; TbL numerous; TbVL numerous when present; TbP numerous, inserted on the caudal margin of the body or on a caudal plate. Ventral ciliation split into two paired longitudinal bands running for the whole length of the body, either reuniting in the cephalic and caudal regions or forming a continuous field covering the pharyngeal region (*M. adenotubulatus*, *M. ischiensis*). Mouth, terminal, opening medium to large; buccal cavity lightly cuticularized, presenting an external hyaline protrusion in *M. ischiensis*. Pharynx bearing pores at the base, opening ventrolaterally; broad anterior intestine, narrowing caudally; anus ventral. Hermaphroditic; ovaries paired; oocytes mature in the caudocephalic direction. Testes elongated, paired, starting near the pharynx‐intestine junction; posteriorly directed vas deferens, discharging directly into the caudal organ. Frontal organ absent; fertilization likely happens through hypodermic impregnation. Interstitial, marine. Included genus: *Mesodasys* Remane, 1951 (type genus).

**Family Redudasyidae** Todaro, Dal Zotto, Jondelius, Hochberg, Hummon, Kanneby and Rocha, 2012

**Emended diagnosis:** Macrodasyidans up to about 1000 μm in total length, occasionally reaching up to 2700 μm (*Dolichodasys*). Body strap‐shaped; head rounded or bluntly triangular; head sensorial structures in the form of several circumcephalic cilia; posterior end rounded, truncated, or two‐lobed, without peduncles. Cuticular covering smooth, without scales or spines. TbA, 1–3 per side: either 1–2 tubes per side, occasionally fused, inserted on short lobes, protruding to the head (*Dolichodasys*, *Paradasys*), or 2–3 tubes of unequal length per side, fused, borne from a common base and emerging from a ventrolateral furrow (*Redudasys*), or inserted in parallel (*Anandrodasys*), protruding obliquely to the rear; TbD absent; TbL/TbVL absent or present, sometimes in the form of short adhesive papillae (*Dolichodasys, P. mahoae*); TbP, present, distributed symmetrically along the caudal margin, or along the caudal lobes; Ventral ciliation split into two paired longitudinal bands along the body, reuniting in an unpaired patch or row caudal to the anus (*Paradasys*, *Redudasys*), or forming a unified field, posterior to the mouth, split in the trunk region into four longitudinal bands, with the two medial bands running along the pharyngeal region and the two lateral bands extending to the caudal region (*Anandrodasys*, *Dolichodasys*). Mouth, terminal or slightly subterminal, narrow; buccal cavity, shallow, lightly cuticularized, sometimes presenting external denticles (*Paradasys*). Pharynx bearing pores at base, opening ventrolaterally. Intestine straight; anus ventral. Ovaries in the hindgut region, paired or central unpaired, with oocytes maturing anteriorly or posteriorly (*Dolichodasys*); male gonad absent in *Anandrodasys*, *Redudasys*, *and* most *Paradasys* species; paired testes in *Dolichodasys*. Frontal organ present in *Dolichodasys*, and occasionally in *Paradasys*, absent in *Anandrodasys* and *Redudasys*; caudal organ present in *Dolichodasys*, absent in *Anandrodasys*, *Paradasys*, and *Redudasys*. Interstitial, marine, or freshwater. Included genera: *Redudasys* Kisielewski, 1987 (type genus); *Anandrodasys* Todaro, Dal Zotto, Jondelius, Hochberg, Hummon, Kånneby and Rocha, 2012; *Dolichodasys* Gagne, 1977; *Paradasys* Remane, 1934.

**Genus *Paradasys*** Remane, 1934

**Emended diagnosis:** Macrodasyidans up to 1000 μm in total length. Body strap‐shaped; head weakly marked, bluntly trapezoidal, often bearing shallow lateral lobes; head sensorial structures in the form of circumcephalic cilia; posterior end truncated or two‐lobed (*P. bilobocaudatus, P. pacificus*), without peduncles. Cuticular covering smooth, without scales or spines, often presenting a granular appearance. TbA 1–2 per side, inserted directly on the ventral body surface or on short lobes; TbD, TbDL, and TbL absent; TbVL absent or short, along the anterior trunk region (*P. mahoae*); TbP from 6 to many, located symmetrically on lateral and posterior borders of the posterior end, separated into groups on either side of the midline, or on lobes. Ventral ciliation split into two paired longitudinal bands along the body, reuniting in an unpaired patch or row caudal to the anus. Mouth, terminal or slightly subterminal, narrow; buccal cavity broadly cylindrical, cuticularized, sometimes presenting external denticles. Pharynx bearing pores at the base, opening ventrolaterally; broad anterior intestine, narrowing caudally; anus ventral. Parthenogenetic or hermaphroditic; ovary single or paired (*P. pacificus*) in the hindgut region; oocytes mature anteriorly. Testes absent in most species; paired testes in *P. lineatus* and *P. littoralis*. Frontal organ, when present, in a caudal position to the oocytes. Interstitial, marine. Included species: *P. subterraneus* Remane, 1934 (type species); *P. bilobocaudus* Hummon, 2008; *P. hexadactylus* Karling, 1954; *P. lineatus* Rao, 1980; *P. littoralis* Rao and Ganapati, 1968; *P. mahoae* (Yamauchi and Kajihara, 2018), comb. nov.; *P. pacificus* Schmidt, 1974.

**Family Urodasyidae fam. nov**.

LSID: urn:lsid:zoobank.org:act:42199BA2‐50BD‐4266‐BC52‐D17775FC177E

**Diagnosis:** Elongate Macrodasyidans, body strap‐shaped, vaulted dorsally and flattened ventrally; head bluntly rounded or oval, weakly marked or not marked from the body; head sensorial structures in the form of circumcephalic cilia, occasionally along with paired piston pits (*Paraurodasys*); the posterior margin ends in a long, filiform, contractile tail, up to three times the length of the body. Cuticular covering smooth, without scales or spines; numerous epidermal glands visible on the whole length of the body, in lateral columns. TbA inserted directly on body surface, in paired diagonal columns or small clusters, sometimes absent (*U. anorektoxys*); TbD/TbDL often present; TbL occurring both in the pharyngeal and trunk region; TbV/TbVL occasionally present; TbP numerous, inserted on the whole length of the tail. Ventral ciliation forms a united field in the pharyngeal region, either continuing uninterrupted for the length of the body (*Urodasys*) or splitting into two paired bands in the trunk region. Mouth, terminal, narrow; buccal cavity shallow, lightly cuticularized. Pharynx bearing pores in the last third, opening ventrolaterally; intestine simple and blind. Hermaphroditic or parthenogenetic; ovaries paired; oocytes mature in the caudocephalic direction; ovoviviparity present in *P. viviparus*. Testes, paired, unpaired, or absent; when present, either elongated, starting near the pharynx‐intestine junction, or short, starting near the caudal region of the intestine; posteriorly directed vas deferens, discharging into a ventral pore. Frontal and caudal organs, present or absent. Interstitial, marine.

Interstitial, marine. Included genera: *Urodasys* Remane, 1926 (type genus); *Paraurodasys*, gen. nov.

**Genus Paraurodasys gen. nov**.

LSID: urn:lsid:zoobank.org:act:ADBCEEB7‐CCFC‐49BD‐BCD0‐088AAF00E3CE

**Etymology:** *Paraurodasys* from the union of “para”, meaning “near to”, plus *Urodasys*, as this new genus is the sister taxon of the established genus *Urodasys* and they share the iconic long tail.

**Diagnosis:** Macrodasyidans up to about 650 μm in total body length, tail excluded. Body strap‐shaped, vaulted dorsally and flattened ventrally; head bluntly rounded or oval, weakly marked or not marked from the body; head sensorial structures in the form of circumcephalic cilia and occasionally paired piston pits; lateral trunk margins can present indentations (*P. bifidostylis, P. poculostylis*); posterior margin ends in a long, filiform, contractile tail, up to three times the length of the body. Cuticular covering smooth, without scales or spines; numerous epidermal glands visible on the whole length of the body, in lateral columns. TbA 3–10 per side, inserted directly on the body surface, in paired diagonal columns or small clusters; TbD/TbDL often present; TbL numerous, occurring both in the pharyngeal and trunk regions; TbV/TbVL occasionally present; TbP numerous, inserted on the whole length of the tail. Ventral ciliation forms a united field in the pharyngeal region, splitting into two paired bands in the trunk region. Mouth, terminal, narrow; buccal cavity shallow, lightly cuticularized. Pharynx bearing pores in the last third, opening ventrolaterally; intestine simple and blind. Hermaphroditic or parthenogenetic (*P. bucinastylis*, *P. viviparus*); ovaries paired; oocytes mature in a caudocephalic direction; ovoviviparity present in *U. viviparus*. Testes paired (*P. completus*) or unpaired, occasionally absent (*P. bucinastylis*, *P. viviparus*); when present, elongated, starting near the pharynx‐intestine junction; posteriorly directed vas deferens, discharging into a ventral pore. Frontal organ, present or absent; when present, posterior to the intestine, muscularized, sac‐like; one external pore, opening dorsally. Caudal organ, when present, a muscular organ containing a sclerotized copulatory stylet with species‐specific conformation, and presenting one external pore, opening ventrally; absent in *P. viviparus*. Interstitial, marine. Included species: *Paraurodasys viviparus* (Wilke, 1954) comb. nov. (type species); *P. acanthostylis* (Fregni, Tongiorgi and Faienza, 1998) comb. nov.; *P. bifidostylis* (Cesaretti et al., 2023) comb. nov.; *P. bucinastylis* (Fregni, Faienza, Grimaldi, Tongiorgi and Balsamo, 1999) comb. nov.; *P. calicostylis* (Schoepfer‐Sterrer, 1974) comb. nov.; *P. completus* (Todaro, Cesaretti and Dal Zotto, 2017) comb. nov.; *P. cornustylis* (Schoepfer‐Sterrer, 1974) comb. nov.; *P. nodostylis* (Schoepfer‐Sterrer, 1974) comb. nov.; *P. poculostylis* (Atherton, 2014) comb. nov.; *P. remostylis* (Schoepfer‐Sterrer, 1974) comb. nov.; *P. spirostylis* (Schoepfer‐Sterrer, 1974) comb. nov.; *P. toxostylus* (Hummon, 2011) comb. nov.; *P. uncinostylis* (Fregni, Tongiorgi and Faienza, 1998) comb. nov.

**Genus *Urodasys*** Remane, 1926

**Emended diagnosis:** Macrodasyidans up to about 1100 μm in total body length, tail excluded. Body strap‐shaped, vaulted dorsally and flattened ventrally; head bluntly rounded or oval, weakly marked or not marked from the body; head sensorial structures in form of circumcephalic cilia; posterior margin ends in a long, filiform, contractile tail, up to three times the length of the body. Cuticular covering smooth, without scales or spines; numerous epidermal glands visible on the whole length of the body, in lateral columns. TbA 4–10 per side, inserted directly on body surface, in paired diagonal columns or small clusters, sometimes absent (*U. anorektoxys*); TbD/TbDL present; TbL numerous, occurring both in the pharyngeal and trunk region; TbV/TbVL occasionally present; TbP numerous, inserted on the whole length of the tail. Ventral ciliation forms a united field in the pharyngeal region, either continuing uninterrupted for the length of the body (*U. anorektoxys*, *U. mirabilis*) or splitting into two paired bands in the trunk region. Mouth, terminal, narrow; buccal cavity shallow, lightly cuticularized. Pharynx bearing pores in the last third, opening ventrolaterally; intestine simple and blind. Hermaphroditic; ovaries paired; oocytes mature in caudocephalic direction. Testes: paired, with the left testes often larger than the other; posteriorly directed vas deferens, discharging into a ventral pore. Frontal and caudal organs absent. Interstitial, marine. Included species: *Urodasys mirabilis* Remane, 1926 (type species); *U. anorektoxys* Todaro, Bernhard and Hummon, 2000; *U. apuliensis* Fregni, Faienza, Grimaldi, Tongiorgi and Balsamo, 1999; *U. elongatus* Renaud‐Mornant, 1969.

## Conflict of interest

The authors declare no conflict of interest.

## Supporting information


**File S1.** Protocol for the validation step.

**Table S1.** Sequences sourced from GenBank for this study, with sampling area, GenBank accession codes and references.

**Table S2.** 18S primers used in the validation step and their respective direction, sequence and usage.

**Appendix S1.** Summary of the nomenclatural acts.

## Data Availability

The data that support the findings of this study are openly available on Figshare under the DOI 10.6084/m9.figshare.30580799, and will be available in GenBank at https://www.ncbi.nlm.nih.gov/genbank/ after publication.
