# Supplementary material for: Improved taxonomic and gene sampling advance the knowledge of deep relationships within Macrodasyida (Gastrotricha)
Source: Cladistics. 2025 Dec 16;42(1):25–45. doi: 10.1111/cla.70013 (PMC12789844; doi:10.1111/cla.70013)
Supplement: Supplementary file 1 — File S1. Protocol for the validation step. [file CLA-42-25-s004.docx]

**File S1. Protocol for the validation step**

PCRs were carried out in a T-Personal thermal cycler (Biometra, Goettingen, Germany). High-fidelity Takara Ex Taq PCR reagents (Takara Bio Inc., Otsu, Japan) were employed following the protocol provided by the manufacturer, using the primer combinations and thermal cycler programs presented in Supplementary Table 2. The PCR products were then purified using the Monarch PCR and DNA Cleanup Kit (New England BioLabs Inc., Ipswich, MA, USA). Sanger sequencing was conducted on the purified products by an external sequencing company (Macrogen Europe Laboratory in Milan, Italy), using the sequencing primers indicated in Supplementary Table 2. The resulting reads were assembled using the Staden package v. 2.0 (Staden, 1996) and the obtained *18S* sequences were examined with the GenBank online BLAST tool (<https://www.ncbi.nlm.nih.gov/genbank/Blast.cgi>).
