# Supplementary material for: Improved taxonomic and gene sampling advance the knowledge of deep relationships within Macrodasyida (Gastrotricha)
Source: Cladistics. 2025 Dec 16;42(1):25–45. doi: 10.1111/cla.70013 (PMC12789844; doi:10.1111/cla.70013)
Supplement: Supplementary file 2 — Table S1. Sequences sourced from GenBank for this study, with sampling area, GenBank accession codes and references. [file CLA-42-25-s002.docx]

**Table S1.** Sequences sourced from GenBank for this study, with sampling area, GenBank accession codes and references.

| **Taxon** | **Sampling area** | **GenBank Accession**  **(18S, 28S, COI)** | **Reference** |
| --- | --- | --- | --- |
| **Cephalodasyidae** |  |  |  |
| *Cephalodasys mahoae*  Yamauchi and Kajihara, 2018 | Hokkaido, Japan  43°15’42” N; 141°21’43” E | LC018992, LC383934, LC383935 | Yamauchi and Kajihara 2018 |
| *Mesodasys laticaudatus* 2 | Tuscany, Italy  42°29’29” N; 11°11’28” E | JF357657, JF357705, JF432043 | Todaro *et al.* 2011 |
| *Mesodasys littoralis* 2 | Bou Ficha, Tunisia  36°16’50” N; 10°29’41” E | JF357658, JF357706, JF432044 | Todaro *et al.* 2011 |
| **Dactylopodolidae** |  |  |  |
| *Dactylopodola mesotyphle*  Hummon, Todaro, Tongiorgi and Balsamo, 1998 | Tuscany, Italy  42°48’42” N; 10°44’46” E | JF357651, JF357699, JF432036 | Todaro *et al.* 2011 |
| *Dactylopodola typhle* 1  (Remane, 1927) | Bou Ficha, Tunisia 36°16’50” N; 10°29’41” E | JF357652, JF357700, JF432037 | Todaro *et al.* 2011 |
| *Dactylopodola typhle* 2 | Tuscany, Italy  42°50’42” N; 10°46’31” E | JF357653, JF357701, JF432038 | Todaro *et al.* 2011 |
| **Macrodasyidae** |  |  |  |
| *Macrodasys* sp. 1 | Tuscany, Italy  42°50’42” N; 10°46’31” E | JF357654, JF357702,  JF432040 | Todaro *et al.* 2011 |
| *Macrodasys* sp. 2 | Bohuslän, Sweden  58°52'05” N; 11°04'57” E | JF357670, JF357714,  JF432052 | Todaro *et al.* 2011 |
| *Urodasys acanthostylis*  Fregni, Tongiorgi and Faienza, 1998 | Lanzarote, Spain  28°55'08” N; 13°40'06” W | PQ415490, PQ429034, PQ462511 | Cesaretti *et al*. 2024 |
| *Urodasys apuliensis*  Fregni, Faienza, Grimaldi, Tongiorgi and Balsamo, 1999 | Sardinia, Italy  41°16’43” N; 09°21’28” E | PQ415491, PQ429035, PQ462513 | Cesaretti *et al*. 2024 |
| *Urodasys completus*  Todaro, Cesaretti and Dal Zotto, 2017 | Lanzarote, Spain  28°55'08” N; 13°40'06” W | PQ415493, PQ429037, PQ462510 | Cesaretti *et al*. 2024 |
| *Urodasys mirabilis*  Remane, 1926 | Willemstad, Curaçao  12°07’19” N; 68°58’09” W | PQ415495, PQ429039, PQ462514 | Cesaretti *et al*. 2024 |
| *Urodasys viviparus*  Wilke, 1954 | Abruzzo, Italy  42°40’44” N; 14°01’05” E | PQ415496, PQ429040, PQ462515 | Cesaretti *et al*. 2024 |
| **Planodasyidae** |  |  |  |
| *Megadasys* sp. 1 | Apulia, Italy  39°50’38” N; 18°23’09” E | JF357655, JF357703, JF432041 | Todaro *et al.* 2011 |
| *Megadasys* sp. 2 | Apulia, Italy  40°15’33” N; 17°53’53” E | JF357656, JF357704, JF432042 | Todaro *et al.* 2011 |
| **Redudasyidae** |  |  |  |
| *Redudasys brasiliensis* 1  Garraffoni, Araújo, Lourenço, Guidi and Balsamo, 2019 | Minas Gerais State, Brazil  18°12′00” S; 43°37′00” W | MH361310, MH361316, MH370134 | Garraffoni *et al*. 2019 |
| *Redudasys brasiliensis* 2 | Minas Gerais State, Brazil  18°12′00” S; 43°37′00” W | MH361311, MH361317, MH370135 | Garraffoni *et al*. 2019 |
| *Redudasys brasiliensis* 3 | Minas Gerais State, Brazil  18°12′00” S; 43°37′00” W | MH361313, MH361319, MH370137 | Garraffoni *et al*. 2019 |
| *Redudasys fornerise*  Kisielewski, 1987 | Sao Paulo, Brazil  22°11’9” S; 47°54’1” W | JN203489, MF577024, KJ950122 | Kånneby and Kirk, 2017 |
| **Thaumatostodermatidae** |  |  |  |
| *Diplodasys meloriae*  Todaro, Balsamo and Tongiorgi, 1992 | Tuscany, Italy  43°33’11” N; 10°13’20” E | JF357640, JF357680, JF432031 | Todaro *et al.* 2011 |
| *Tetranchyroderma hirtum*  Luporini, Magagnini and Tongiorgi, 1973 | Tuscany, Italy  43°00’53” N; 09°49’24” E | JF357628, JF357676, JF432023 | Todaro *et al.* 2011 |
| *Oregodasys tentaculatus*  (Swedmark, 1956) | Tuscany, Italy  43°33’11” N; 10°13’20” E | JF357626, JF357674, JF432021 | Todaro *et al.* 2011 |
| *Pseudostomella etrusca*  Hummon, Todaro and Tongiorgi, 1993 | Tuscany, Italy  42°29’29” N; 11°11’28” E | JF357633, JF357681, JF432026 | Todaro *et al.* 2011 |
| *Ptychostomella tyrrhenica*  Hummon, Todaro and Tongiorgi, 1993 | Tuscany, Italy  42°29’29” N; 11°11’28” E | JF357634, JF357682, JF432027 | Todaro *et al.* 2011 |
| **Turbanellidae** |  |  |  |
| *Paraturbanella pallida*  Luporini, Magagnini and Tongiorgi, 1973 | Tuscany, Italy  43°00’53” N; 09°49’24” E | JF357660, JF357708, JF432045 | Todaro *et al.* 2011 |
| *Turbanella bocqueti*  Kaplan, 1958 sensu Boaden, 1974 | Waterford, Ireland  52°09’24” N; 07°08’12” W | JF357662, JF357710, JF432046 | Todaro *et al.* 2011 |
| *Turbanella cornuta*  Remane, 1925 | Veneto, Italy  45°12’57” N; 12°17’57” E | JF357663, JF357711, JF432047 | Todaro *et al.* 2011 |
| **Muselliferidae** |  |  |  |
| *Diuronotus aspetos*  Todaro, Balsamo and Kristensen, 2005 | Disko Island, Greenland  69°38’63” N; 51°51’13” W | KX531005, KX531006, KX531007 | Bekkouche and Worsaae, 2016 |
| **Xenotrichulidae** |  |  |  |
| *Xenotrichula intermedia*  Remane, 1934 | Mahdia, Tunisia  35°30’57” N; 11°03’00” E | JF357664, JF357712, JF432948 | Todaro *et al.* 2011 |
