# Supplementary material for: Improved taxonomic and gene sampling advance the knowledge of deep relationships within Macrodasyida (Gastrotricha)
Source: Cladistics. 2025 Dec 16;42(1):25–45. doi: 10.1111/cla.70013 (PMC12789844; doi:10.1111/cla.70013)
Supplement: Supplementary file 3 — Table S2. 18S primers used in the validation step and their respective direction, sequence and usage. [file CLA-42-25-s001.docx]

Table S2. 18S primers used in the validation step and their respective direction, sequence and usage.

| **Primer** | **Direction** | **Sequence 5’ to 3’** | **Usage** | **Reference** |
| --- | --- | --- | --- | --- |
| S30 | forward | GCTTGTCTCAAAGATTAAGCC | PCR | Norén and Jondelius, 1999 |
| 1806R | reverse | CCTTGTTACGACTTTTACTTCCTC | PCR | Norén and Jondelius, 1999 |
| PCR regime | 3 min at 95 °C, 35 × (30 s at 94 °C, 30 s at 50 °C, 2 min at 72 °C), 7 min at 72 °C | | | |
| 18S R536 | reverse | CTGGAATTACCGCGGCTG | sequencing | Rosati et al., 2004 |
| 18S R1052 | reverse | AACTAAGAACGGCCATGCA | sequencing | Rosati et al., 2004 |
| 18S F783 | forward | GACGATCAGATACCGTC | sequencing | Rosati et al., 2004 |
