# Supplementary material for: Improved taxonomic and gene sampling advance the knowledge of deep relationships within Macrodasyida (Gastrotricha)
Source: Cladistics. 2025 Dec 16;42(1):25–45. doi: 10.1111/cla.70013 (PMC12789844; doi:10.1111/cla.70013)
Supplement: Supplementary file 4 — Appendix S1. Summary of the nomenclatural acts. [file CLA-42-25-s003.docx]

# SUMMARY OF THE NOMENCLATURAL ACTS

1. Establishment of Mesodasyidae fam. nov. (reassignment of *Mesodasys* from Cephalodasyidae to Mesodasyidae fam. nov.)

2. Establishment of Urodasyidae fam. nov. (reassignment of *Urodasys* from Macrodasyidae to Urodasyidae fam. nov.)

3. Establishment of *Paraurodasys* gen. nov.,

4. Reassigment of *Urodasys acanthostylis,* *U. bifidostylis,* *U. bucinastylis,* *U. calicostylis,* *U. completus,* *U. cornustylis,* *U. nodostylis,* *U. poculostylis,* *U. remostylis,* *U. spirostylis,* *U. toxostylus,* *U. uncinostylis,* *U. viviparus* to *Paraurodasys* gen. nov.

5. Reassignment of *Cephalodasys mahoae* to genus *Paradasys*

6. Reassignment of genus *Paradasys* from Cephalodasyidae to Redudasyidae

7. Reassignment of genus *Dolichodasys* from Cephalodasyidae to Redudasyidae

See details of all the nomenclatural acts in the section below.

# DIAGNOSES

Order Macrodasyida Remane, 1925 [Rao and Clausen, 1970]

**Family Cephalodasyidae** Hummon and Todaro, 2010 emend.

**Emended diagnosis:** Elongate Macrodasyidans up to 1800 μm in total length. Body strap-shaped, flattened ventrally and vaulted dorsally; head rounded, marked by a posterior constriction; head sensorial structures in form of circumcephalic cilia; posterior end rounded, broadly expanded or tapering into a medial process. Cuticular covering smooth, without scales or spines; epidermal glands often present. TbA in two groups, inserting on fleshy “hands”; lateral, dorsolateral and ventrolateral adhesive tubes (TbL/TbDL/TbVL) arranged in columns along the body; posterior adhesive tubes (TbP) arranged marginally around the posterior end. Ventral ciliation split into 2 paired longitudinal bands along the body, reuniting caudally. Mouth, terminal or slightly subterminal, narrow; buccal cavity broadly cylindrical, lightly cuticularized. Sphincter muscle developed around mouth opening; well-developed striated radial pharyngeal musculature. Circular muscles present in lateral regions of the body. Y-cells absent. Pharynx bearing pores at base, opening ventrolaterally; broad anterior intestine, narrowing caudally. Hermaphroditic; ovary single, central, oocytes maturing posterior to anterior (*Pleurodasys*) or anterior to posterior (*Cephalodasys*). Testes paired, male gametes mature posterior to anterior. Frontal and caudal organ present in *Cephalodasys*; absent in *Pleurodasys*. Interstitial, marine. Type genus: *Cephalodasys* Remane, 1926. Other genus: *Pleurodasys* Remane, 1927.

**Family Macrodasyidae** Remane, 1924 emend.

**Emended diagnosis:** Elongate Macrodasyidans up to 1000 μm in total length. Body strap-shaped, elongated; head bluntly rounded or ovoid, sometimes marked by a posterior constriction (*Kryptodasys*, *Thaidasys*); head sensorial structures in form of circumcephalic cilia and pestle organs, or leaf-like organs (*Thaidasys*); posterior end ovoidal or tapering, sometimes in form of a short tail (*Macrodasys*). Cuticular covering smooth, without scales or spines; epidermal glands either inconspicuous or visible (*Thaidasys*). TbA 2-7 per side, inserted directly on body surface in diagonal columns or short arcs; lateral and ventrolateral adhesive tubes (TbL/TbVL) arranged in columns along the body; TbD sometimes present (*Thaidasys*); posterior adhesive tubes (TbP) arranged marginally around the posterior end or along the tail. Ventral ciliation either split into 2 paired longitudinal bands along the body (*Thaidasys*), or forming a single field that can split into 2 paired longitudinal bands along the caudal region. Mouth, terminal, medium size; buccal cavity lightly cuticularized, shallow. Pharynx bearing pores significantly anterior of the pharyngo-intestinal junction, opening ventrolaterally; intestine straight, sometimes broader in the middle section (*Kryptodasys*). Hermaphroditic; ovary single, oocytes maturing posterior to anterior. Testes paired, elongated, starting near the pharyngo-intestinal junction, or absent (*Thaidasys*). Frontal organ, present or absent (*Thaidasys*); caudal organ present as a muscular organ containing a copulatory tube or canal. Interstitial, marine. Type genus: *Macrodasys* Remane, 1924. Other genera: *Kryptodasys* Todaro, Dal Zotto, Kånneby and Hochberg, 2019; *Thaidasys* Todaro, Dal Zotto and Leasi, 2015.

**Family Mesodasyidae fam. nov.**

LSID: urn:lsid:zoobank.org:act:53A43C3C-92C2-446D-8CD9-BECC3F9172B6

**Diagnosis:** Same as the genus.

Interstitial, marine. Includes genus *Mesodasys* Remane, 1951*.*

**Genus *Mesodasys*** Remane, 1951

**Diagnosis:** Macrodasyidans up to 2000 μm in total length. Body strap-shaped, elongated; head bluntly rounded or truncated, unmarked from the body; head sensorial structures in form of circumcephalic cilia; posterior end rounded, tapering or ending in a rounded caudal lobe. Cuticular covering smooth, without scales or spines; numerous epidermal glands visible for the whole length of the body, in lateral columns. TbA numerous, inserted directly on body surface in transverse rows, diagonal columns or a single uninterrupted arc following the anterior profile; TbL numerous; TbD and TbVL numerous when present,; TbP numerous, inserted on the caudal margin of the body or on a caudal plate. Ventral ciliation split into 2 paired longitudinal bands running for the whole length of the body, either reuniting in the cephalic and caudal region or forming a continuous field covering the pharyngeal region (*M. adenotubulatus*, *M. ischiensis*). Mouth, terminal, opening medium to large; buccal cavity lightly cuticularized, presenting an external hyaline protrusion in *M. ischiensis*. Pharynx bearing pores at base, opening ventrolaterally; broad anterior intestine, narrowing caudally. Hermaphroditic; ovaries paired; oocytes mature in caudo-cephalic direction. Testes elongated, paired, starting near the pharynx-intestine junction; posteriorly directed sperm ducts, discharging directly into the caudal organ. Frontal organ not recorded in any described species, fertilization likely happens through hypodermic impregnation. Interstitial, marine. Includes species: *Mesodasys adenotubulatus* Hummon, Todaro and Tongiorgi, 1993; *Mesodasys brittanica* Hummon, 2008; *Mesodasys hexapodus* Rao and Ganapati, 1968; *Mesodasys ischiensis* Hummon, Todaro and Tongiorgi, 1993; *Mesodasys laticaudatus* Remane, 1951; *Mesodasys littoralis* Remane, 1951; *Mesodasys rupperti* Hummon, 2008; *Mesodasys saddlebackensis* Hummon, 2010.

**Family Redudasyidae** Todaro, Dal Zotto, Jondelius, Hochberg, Hummon, Kanneby and Rocha, 2012 emend.

**Emended diagnosis:** Macrodasyidans up to about 1000 μm in total length, occasionally reaching up to 2700 μm (*Dolichodasys*). Body strap-shaped; head rounded or bluntly triangular; head sensorial structures in form of several circumcephalic cilia; posterior end rounded, truncated or two-lobed, without peduncles. Cuticular covering smooth, without scales or spines. Adhesive apparatus mainly consisting of anterior (TbA) and posterior tubes (TbP); lateral (TbL) or ventrolateral tubes (TbVL) may also be present in form of short adhesive papillae (*Anandrodasys*, *Paradasys*). TbA, 1–3 per side: either 1–2 tubes per side, occasionally fused, inserted on short lobes, protruding to the head (*Dolichodasys*, *Paradasys*), or 2–3 tubes of unequal length per side, fused, borne from a common base and emerging from a ventrolateral furrow (*Redudasys*), or inserted in parallel (*Anandrodasys*), protruding obliquely to the rear. TbVL, if present, 5–6 per side, along the anterior intestinal region. Dorsal tubes (TbD) absent. TbP, 4–12 in total, distributed symmetrically on the caudal margin, separated into groups on either side of midline (*Dolichodasys*, *Paradasys*), or at the end of two caudal lobes (*Anandrodasys*, *Redudasys*). Ventral ciliation split into 2 paired longitudinal bands along the body, reuniting in an unpaired patch or row caudal to the anus (*Paradasys*, *Redudasys*), or forming a unified field, posterior to the mouth, split in the trunk region into 4 longitudinal bands, with the 2 medial bands running along the pharyngeal region and the 2 lateral bands extending to the caudal region (*Anandrodasys*, *Dolichodasys*). Mouth, terminal or slightly subterminal, narrow; buccal cavity, shallow, lightly cuticularized, sometimes presenting external denticles (*Paradasys*). Pharynx bearing pores at base, opening ventrolaterally. Intestine straight; anus ventral. Ovaries in hindgut region, paired or central unpaired, with oocytes maturing anteriorly or posteriorly (*Dolichodasys*); male apparatus unknown in *Anandrodasys*, *Redudasys,* most *Paradasys* species; paired testes in *Dolichodasys*. Frontal and caudal organs absent in *Anandrodasys*, *Redudasys*, present in *Dolichodasys*. Interstitial, marine or freshwater. Type genus: *Redudasys* Kisielewski, 1987. Other genera: *Anandrodasys* Todaro, Dal Zotto, Jondelius, Hochberg, Hummon, Kånneby and Rocha, 2012; *Dolichodasys* Gagne, 1977; *Paradasys* Remane, 1934.

**Family Redudasyidae** Todaro, Dal Zotto, Jondelius, Hochberg, Hummon, Kanneby and Rocha, 2012

**Genus *Paradasys*** Remane, 1934 emend.

**Emended diagnosis:** Macrodasyidans up to 1000 μm in total length. Body strap shaped; head weakly marked, bluntly trapezoidal, often bearing shallow lateral lobes; head sensorial structures in form of circumcephalic cilia; posterior end truncated or two-lobed (*P. bilobocaudatus, P. pacificus*), without peduncles. Cuticular covering smooth, without scales or spines, often presenting a granular appearance. TbA 1-2 per side, inserted directly on ventral body surface or on short lobes; TbL absent or short (4-6 μm in length) and present in the anterior trunk region (*P. mahoae*); TbP from 6 to many, located symmetrically on lateral and posterior borders of posterior end, separated into groups on either side of midline or on lobes. Ventral ciliation split into 2 paired longitudinal bands along the body, reuniting in an unpaired patch or row caudal to the anus. Mouth, terminal or slightly subterminal, narrow; buccal cavity broadly cylindrical, cuticularized, sometimes presenting external denticles. Pharynx bearing pores at base, opening ventrolaterally; broad anterior intestine, narrowing caudally. Parthenogenetic or hermaphroditic; ovary single or paired (*P. pacificus*), in the hindgut region; oocytes mature anteriorly. Testes absent in most species; paired testes in *P. lineatus*, *P. littoralis*. Frontal organ, when present, in caudal position to the oocytes. Interstitial, marine. Includes species: *Paradasys bilobocaudus* Hummon, 2008; *Paradasys hexadactylus* Karling, 1954; *Paradasys lineatus* Rao, 1980; *Paradasys littoralis* Rao and Ganapati, 1968; ***Paradasys mahoae*** (Yamauchi and Kajihara, 2018), **comb. nov.**; *Paradasys pacificus* Schmidt, 1974; *Paradasys subterraneus* Remane, 1934.

**Family Urodasyidae fam. nov.**

LSID: urn:lsid:zoobank.org:act:42199BA2-50BD-4266-BC52-D17775FC177E

**Diagnosis:** Elongate Macrodasyidans, body strap-shaped, vaulted dorsally and flattened ventrally; head bluntly rounded or oval, weakly marked or not marked from the body; head sensorial structures in form of circumcephalic cilia, occasionally along with paired piston pits (*Paraurodasys*); the posterior margin ends in a long, filiform, contractile tail, up to three times the length of the body. Cuticular covering smooth, without scales or spines; numerous epidermal glands visible on the whole length of the body, in lateral columns. TbA inserted directly on body surface, in paired diagonal columns or small clusters, sometimes absent (*U. anorektoxys*); TbL occurring both in the pharyngeal and trunk region; TbD and TbV occasionally present; TbP numerous, inserted on the whole length of the tail. Ventral ciliation forms a united field in the pharyngeal region, either continuing uninterrupted for the length of the body (*Urodasys*) or splitting into two paired bands in the trunk region. Mouth, terminal, narrow; buccal cavity shallow, lightly cuticularized. Pharynx bearing pores in the last third, opening ventrolaterally; intestine simple and blind. Hermaphroditic or parthenogenetic (*Paraurodasys*); ovaries paired; oocytes mature in caudo-cephalic direction; ovoviviparity present in *P. viviparus*. Testes, paired, unpaired, or absent; when present, either elongated, starting near the pharynx-intestine junction, or short, starting near the caudal region of the intestine; posteriorly directed sperm ducts, discharging into a ventral pore. Frontal and caudal organs, present or absent. Interstitial, marine.

Interstitial, marine. Type genus: *Urodasys* Remane, 1926. Other genus: ***Paraurodasys*, gen. nov.**

**Family Urodasyidae fam. nov.**

**Genus *Urodasys*** Remane, 1926 emend.

**Emended diagnosis:** Macrodasyidans up to about 1100 μm in total body length, tail excluded. Body strap-shaped, vaulted dorsally and flattened ventrally; head bluntly rounded or oval, weakly marked or not marked from the body; head sensorial structures in form of circumcephalic cilia; posterior margin ends in a long, filiform, contractile tail, up to three times the length of the body. Cuticular covering smooth, without scales or spines; numerous epidermal glands visible on the whole length of the body, in lateral columns. TbA 4 to 10 per side, inserted directly on body surface, in paired diagonal columns or small clusters, sometimes absent (*U. anorektoxys*); TbL numerous, occurring both in the pharyngeal and trunk region; TbD and TbV occasionally present; TbP numerous, inserted on the whole length of the tail. Ventral ciliation forms a united field in the pharyngeal region, either continuing uninterrupted for the length of the body (*U. anorektoxys*, *U. mirabilis*) or splitting into two paired bands in the trunk region. Mouth, terminal, narrow; buccal cavity shallow, lightly cuticularized. Pharynx bearing pores in the last third, opening ventrolaterally; intestine simple and blind. Hermaphroditic; ovaries paired; oocytes mature in caudo-cephalic direction. Testes, paired, with the left testes often larger than the other; posteriorly directed sperm ducts, discharging into a ventral pore. Frontal and caudal organs absent. Interstitial, marine.

Includes species: *Urodasys anorektoxys* Todaro, Bernhard and Hummon, 2000; *Urodasys apuliensis* Fregni, Faienza, Grimaldi, Tongiorgi and Balsamo, 1999; *Urodasys elongatus* Renaud-Mornant, 1969; *Urodasys mirabilis* Remane, 1926.

**Genus *Paraurodasys* gen. nov.**

LSID: urn:lsid:zoobank.org:act:ADBCEEB7-CCFC-49BD-BCD0-088AAF00E3CE

**Etymology:** *Paraurodasys* from the union of “para”, meaning “near to”, plus *Urodasys*, as this new genus is the sister taxon of the established genus *Urodasys* and they share the iconic long tail.

**Type species:** *Paraurodasys viviparus* (Wilke, 1954) comb. nov.

**Diagnosis:** Macrodasyidans up to about 650 μm in total body length, tail excluded. Body strap-shaped, vaulted dorsally and flattened ventrally; head bluntly rounded or oval, weakly marked or not marked from the body; head sensorial structures in form of circumcephalic cilia and occasionally paired piston pits; lateral trunk margins can present indentations (*P. bifidostylis, P. poculostylis*); posterior margin ends in a long, filiform, contractile tail, up to three times the length of the body. Cuticular covering smooth, without scales or spines; numerous epidermal glands visible on the whole length of the body, in lateral columns. TbA 3 to 10 per side, inserted directly on body surface, in paired diagonal columns or small clusters; TbL numerous, occurring both in the pharyngeal and trunk region; TbD and TbV occasionally present; TbP numerous, inserted on the whole length of the tail. Ventral ciliation forms a united field in the pharyngeal region, splitting into two paired bands in the trunk region. Mouth, terminal, narrow; buccal cavity shallow, lightly cuticularized. Pharynx bearing pores in the last third, opening ventrolaterally; intestine simple and blind. Hermaphroditic or parthenogenetic (*P. bucinastylis*, *P. viviparus*); ovaries paired; oocytes mature in caudo-cephalic direction; ovoviviparity present in *P. viviparus*. Testes paired (*P. completus*) or unpaired, occasionally absent (*P. bucinastylis*, *P. viviparus*); when present, elongated, starting near the pharynx-intestine junction; posteriorly directed sperm ducts, discharging into a ventral pore. Frontal organ, present or absent; when present, posterior to the intestine, muscolarized, sac-like; one external pore, opening dorsally. Caudal organ, occasionally absent (*P. viviparus*); when present, a muscular organ containing a sclerotized copulatory stylet with species-specific conformation; one external pore, opening ventrally.

Interstitial, marine. Includes species: ***Paraurodasys acanthostylis*** (Fregni, Tongiorgi and Faienza, 1998) **comb. nov.**; ***Paraurodasys bifidostylis*** (Cesaretti, Leasi and Todaro, 2023) **comb. nov.**; ***Paraurodasys bucinastylis*** (Fregni, Faienza, Grimaldi, Tongiorgi and Balsamo, 1999) **comb. nov.**; ***Paraurodasys calicostylis*** (Schoepfer-Sterrer, 1974) **comb. nov.**; ***Paraurodasys completus*** (Todaro, Cesaretti and Dal Zotto, 2017) **comb. nov.**; ***Paraurodasys cornustylis*** (Schoepfer-Sterrer, 1974) **comb. nov.**; ***Paraurodasys nodostylis*** (Schoepfer-Sterrer, 1974) **comb. nov.**; ***Paraurodasys poculostylis*** (Atherton, 2014) **comb. nov.**; ***Paraurodasys remostylis*** (Schoepfer-Sterrer, 1974) **comb. nov.**; ***Paraurodasys spirostylis*** (Schoepfer-Sterrer, 1974) **comb. nov.**; ***Paraurodasys toxostylus*** (Hummon, 2011) **comb. nov.**; ***Paraurodasys uncinostylis*** (Fregni, Tongiorgi and Faienza, 1998) **comb. nov.**; ***Paraurodasys viviparus*** (Wilke, 1954) **comb. nov.**
